# Impact of accurate load forecasting on electricity market stability in Japan using classical time-series and deep-learning methods

**DOI:** 10.1038/s41598-026-46859-2

**Published:** 2026-04-02

**Authors:** Dalia Rabie, Mehran Moradi, Wang Xuan, Hooman Farzaneh

**Affiliations:** 1 Interdisciplinary Graduate School of Engineering Sciences, Kyushu University, Fukuoka, 816-8580 Japan; 2 Transdisciplinary Research and Education Center for Green Technologies, Kyushu University, Fukuoka, Japan; 3 Faculty of Engineering Sciences, Kyushu University, Fukuoka, Japan; 4 Department of Engineering and Physics, Karlstad University, Karlstad, 651 88 Sweden

**Keywords:** Short-term load forecasting, SARIMA, Hidden Markov model, Long short-term memory, Japan electric power exchange, Climate sciences, Mathematics and computing

## Abstract

This study presents a novel multi-regional evaluation framework for short-term load forecasting (STLF) in Japan’s structurally fragmented, regionally heterogeneous electricity market. While previous research often treats the Japanese grid as a monolith or focuses on a single forecasting paradigm, this study addresses the critical research gap of spatial performance variability across the 50/60 Hz frequency divide. Motivated by the critical need for regional accuracy, this research compares three forecasting paradigms: the classical SARIMA model, the probabilistic Hidden Markov Model (HMM), and the deep learning Long Short-Term Memory (LSTM) network. Using hourly load data from 2019 to 2022 for all nine Japanese regional power systems, the models are evaluated across two horizons (day-ahead and hour-ahead. Furthermore, an Uncertainty Quantification (UQ) framework is employed to generate 95% Prediction Intervals (PI) under extreme operating conditions—including maximum demand, minimum demand, and public holidays—in order to assess model robustness under atypical scenarios. Results show that no single model is universally superior; forecasting performance is highly context and region-dependent. In Tokyo on a maximum demand day, LSTM achieves the best accuracy (4.55% MAPE), outperforming SARIMA (6.31% MAPE). In contrast, for Chugoku under the same scenario, SARIMA (2.72% MAPE) slightly outperforms LSTM (2.80% MAPE). The HMM proved particularly effective for atypical conditions, delivering the most accurate forecast in Tohoku on a public holiday (4.03% MAPE versus 6.93% for SARIMA). The findings underscore the critical impact of statistical forecasting errors on regional financial risk. This framework reveals that improved accuracy can reduce daily financial burdens by 5.4 million yen in Chugoku, 102 million yen in Tohoku, and up to 642 million yen in Tokyo. By linking forecasting accuracy, uncertainty, and economic impact, the proposed framework provides practical guidance for context-aware forecasting strategies in Japan’s fragmented electricity market.

**Supplementary Information:**

The online version contains supplementary material available at 10.1038/s41598-026-46859-2.

## Introduction

### Background and motivation

The global electricity sector has experienced a profound transformation, evolving from regulated monopolies into liberalized, competitive markets. This paradigm shift has redefined electricity as a tradable commodity, where supply and demand are matched through transparent, market-based mechanisms^1^. In line with global liberalization trends, the day-ahead electricity market has become the primary platform for wholesale electricity trading. In this market, the Market Clearing Price (MCP) is determined through a double-blind auction process that balances supply and demand^2^. This mechanism provides financial and operational certainty, allowing market participants to hedge against price volatility and secure energy procurement in advance.

However, when a region’s local generation is insufficient to meet its demand, cross-regional interconnections are activated through the spot market under day-ahead contracts. In such cases, the regional MCP replaces the national MCP according to the implicit auction policy, which prioritizes local balancing of supply and demand unless external supply is procured through the spot market^3^. Therefore, the efficiency and reliability of this market structure fundamentally depend on the accuracy of load forecasting. Accurate forecasts maximize profit and minimize cost for market participants while ensuring grid stability for system operators. Inaccurate predictions can result in severe economic and operational consequences: over-forecasting leads to unnecessary purchases, while under-forecasting causes shortages that must be covered in real-time markets—typically more volatile and expensive. Thus, load forecasting forms the foundation of operational planning and market stability in modern power systems^4^. In this regard, a broad spectrum of forecasting methodologies has been developed and employed, ranging from classical statistical models such as Seasonal Auto-Regressive Integrated Moving Average (SARIMA) to advanced machine and deep learning approaches, including Long Short-Term Memory (LSTM) and hybrid neural architectures^5,6^.

The Japan Electric Power Exchange (JEPX), as one of the leading day-ahead wholesale electricity markets in the world, provides a prosperous and complex case study for examining load forecasting methodologies in a liberalized electricity market. Although Japan completed full retail electricity liberalization in 2016, its electricity system remains structurally fragmented across nine regional service areas^7^. The grid is further divided by a 50 Hz/60 Hz frequency split between Eastern and Western Japan, with limited interconnection capacity between regions. This physical segmentation prevents JEPX from functioning as a single, unified market. Instead, distinct regional prices are cleared for each service area, reflecting differences in demand patterns shaped by climate, industrial composition, population density, and lifestyle behavior. For instance, northern regions exhibit high winter heating loads, whereas southern regions experience summer peaks due to air conditioning^8^.

Such structural fragmentation poses significant challenges for electricity demand forecasting. Demand dynamics differ substantially across regions due to variations in climatic, socioeconomic, and consumption characteristics. Consequently, relying on a single aggregated national forecasting model may obscure critical regional differences in demand behavior and forecasting performance.

Although a substantial body of research exists on short-term load forecasting (STLF), existing studies in the Japanese context often focus on either aggregated datasets or single-system case studies. There is a notable research gap in understanding how different forecasting architectures—classical time-series, probabilistic, and deep learning—perform across geographically and operationally diverse regions under extreme demand conditions. This study addresses this gap by presenting a comprehensive comparative framework that tests model resilience across nine regions and various atypical demand scenarios, ultimately linking technical accuracy to regional economic stability.

### Literature survey

Accurate load forecasting plays a vital role in maintaining grid stability, optimizing generation scheduling, and supporting efficient market operations in liberalized electricity systems. Over the past two decades, the evolution of forecasting methodologies has reflected the growing complexity of modern power systems — moving from classical statistical models to deep learning frameworks capable of capturing non-linear temporal dependencies^9^. Among traditional time series models, the SARIMA model remains one of the most widely applied techniques for electricity demand forecasting due to its transparency, interpretability, and strong ability to capture periodic and seasonal components. Al-Shaikh et al. (2019) demonstrated that SARIMA delivers reliable short-term forecasts when seasonal patterns are stable and consistent, often outperforming baseline approaches^10^. Andoh et al. (2021) specifically highlighted the model’s interpretability and transparency in system operation^11^. Kien et al. (2023) demonstrated SARIMA’s strong performance and “robustness” in the emerging market of Hanoi, Vietnam, confirming its ability to “ensure forecast accuracy” in systems characterized by clear and stable seasonal cycles^12^. However, the model’s performance is critically dependent on the assumption of linearity and stationary seasonality. Its limitations become apparent when faced with high volatility and non-linear dynamics, as noted in an analysis of the Turkish Electricity Market, which stated the model’s performance decline under such highly dynamic load conditions^13^. This finding is echoed in 2025 research, which highlights SARIMA’s inherent limitations when dealing with systems characterised by pronounced nonlinearities^14^. However, SARIMA continues to serve as an indispensable benchmark model in operational contexts due to its low computational cost and ease of implementation — particularly in regions where electricity demand exhibits strong periodicity, or seasonally consistent systems^15^.

To address uncertainty and non-stationarity in modern power systems, researchers have explored probabilistic approaches, representing a bridge between classical and modern paradigms with a distinct paradigm that introduces probabilistic state-based modelling into the forecasting process, among these, the Hidden Markov Model (HMM)^16^. Unlike SARIMA, which assumes continuous and stationary relationships, HMMs are capable of capturing discrete regime changes, making them particularly useful for identifying structural breaks or shifts in operational states. Early applications by Zhang et al. (2010) established the potential of HMMs in modelling the stochastic, state-dependent behaviour of electricity prices^17^. Erlwein et al. (2010) applied HMMs to model the stochastic, regime-switching dynamics of electricity spot prices in the Nord Pool market, demonstrating the model’s strength in capturing state-dependent behavior^18^. This approach was later extended to consumption data. In a key study, Ullah et al. (2018) used HMMs to model household energy consumption using high-frequency data from residential buildings in Seoul, South Korea^19^. The authors demonstrated that the HMM could effectively model the behavioral variability and uncertainty in noisy data, achieving superior performance compared to other machine learning models like Artificial Neural Networks (ANN) and Support Vector Machines (SVM) in their specific context. More recently, Khetarpal et al. (2023) found that HMMs can achieve accuracy comparable to neural networks when data availability is limited, highlighting their practicality in data-sparse contexts^20^. In the context of STLF, HMMs are particularly useful for identifying structural breaks or shifts in operational states, such as the distinct transition between a regular weekday consumption pattern and an anomalous public holiday pattern. Thus, HMM represents a specialized paradigm focused on state-based, stochastic transitions rather than continuous temporal dependencies.

Recent advances in deep learning have substantially reshaped STLF, with the Long Short-Term Memory (LSTM) neural network emerging as one of the most powerful models for capturing complex temporal dependencies and long-range correlations in electricity demand^21^. LSTM, a type of recurrent neural network (RNN), is explicitly designed to overcome the limitations of traditional models by capturing highly complex, non-linear temporal dependencies and long-range correlations in sequential data^2^. Kong et al. (2017) first demonstrated that LSTM architectures outperform both traditional statistical models and other machine learning methods on smart meter data, achieving lower Root Mean Square Error (RMSE) and Mean Absolute Percentage Error (MAPE) in both hour-ahead and day-ahead horizons^22^. Subsequent work by Miah et al. (2025) developed an LSTM-based deep learning model (REDf) and evaluated it using “four historical short-term energy demand datasets” from different U.S. utility companies. The study found “superior performance shown that LSTM configurations maintain high forecasting accuracy and generalization across multiple regional datasets^23^. Separately, Ahranjani et al. (2025) have shown that hybrid deep learning configurations also maintain high accuracy across diverse operating conditions^24^. LSTM’s ability to dynamically learn from past information and model high-dimensional, non-linear interactions makes it especially effective in volatile and complex demand environments, such as large urban centers with diverse load components^25^. This superior performance, however, often comes at the cost of greater computational complexity and a “black box” nature, reducing its interpretability compared to classical models like SARIMA^26,27^. Recent studies have increasingly explored the nuances of adaptive forecasting and integrated operational strategies in volatile energy environments. Methodologically, advancements in deep learning have proven highly effective in capturing complex nonlinear patterns; for instance, Kırat et al. (2024) demonstrated the utility of architectures like N-HiTS and LSTM for market price prediction and battery arbitrage^28^, while Çiçek et al. (2024) applied the Time-series Dense Encoder (TiDE) model to forecast PV generation and electricity prices, enabling efficient hydrogen demand management and electricity trading, which improved operational flexibility and generated €1,144.84 in real-time revenue despite lower PV output^29^.

Beyond pure forecasting, the literature has shifted toward the strategic deployment of these models within modern grid architectures. Nguyen Hong and Nguyen Duc (2024) explored the strategic decision-making of Virtual Power Plants (VPPs) integrating energy storage in day-ahead and balancing markets, highlighting how handling predictive uncertainty directly informs optimal scheduling and bidding efficiency^30^. Similarly, El-Azab et al. (2025) note that the operational reliability of emerging technologies like smart grids and electric vehicles under variable weather and temperature conditions relies heavily on seasonal forecasting of the hourly electricity demand^31^.

In the specific context of the Japanese electricity market (JEPX), Jiang and Yamada (2025) applied machine learning models such as LightGBM and Random Forest to predict imbalance signals, highlighting the significant influence of weather-related variables and generator outages on supply–demand stability. The study also showed that incorporating regionally diverse meteorological data improves forecasting accuracy^32^. Building on these insights, this study adopts a multi-regional uncertainty quantification (UQ) framework to evaluate forecasting performance across Japan’s fragmented electricity market.

Although SARIMA, HMM, and LSTM have each been widely applied in short-term load forecasting studies, most prior research evaluates these models independently or compares them only with methods from the same methodological family. Consequently, comprehensive cross-paradigm evaluations involving statistical, probabilistic, and deep learning approaches within a unified experimental framework remain relatively limited. This limitation makes it difficult to fully understand the relative strengths and weaknesses of these fundamentally different forecasting paradigms when applied to identical datasets and operational conditions. Furthermore, existing studies rarely consider the broader operational and market contexts in which forecasting models are applied, as multi-regional comparisons, market implications of forecasting errors, and model robustness under atypical demand scenarios remain insufficiently explored.

The past studies summarized in Table 1 reveal a clear consensus: no single model is universally optimal. A SARIMA model may outperform a complex neural network in stable systems, whereas an LSTM network may perform better under highly nonlinear and volatile conditions. In other words, forecasting performance largely depends on the alignment between a model’s architecture and the statistical characteristics of the dataset. However, the practical implications of this observation to provide empirical proof that the optimal forecasting strategy is a function of regional demographic characteristics remain insufficiently addressed in the literature.

### Research gaps and study contributions

Although previous studies generally acknowledge that no single forecasting model consistently outperforms others across all datasets and operating conditions, the practical implications of this observation remain insufficiently addressed in the literature. Specifically, there is a lack of systematic investigations that determine which forecasting paradigm performs best under particular regional characteristics, demand conditions, and forecasting horizons, especially within fragmented electricity markets characterized by strong regional heterogeneity. As a result, electricity market participants often lack clear guidance on how to select forecasting models that are most appropriate for specific operational contexts.

Despite the extensive literature on STLF, ranging from SARIMA to advanced deep learning architectures, existing literature frequently overlooks several important gaps :

- First, multi-regional comparative analyses remain scarce, particularly in fragmented power systems such as Japan’s, where regional heterogeneity and the 50/60 Hz divide are often overlooked. Most existing studies focus on individual regions or aggregated national datasets, which can obscure regional differences in demand behavior.
- Second, most research focuses primarily on statistical accuracy metrics while neglecting the market and financial implications of forecasting errors in competitive environments like JEPX. This creates a gap between methodological forecasting research and the practical decision-making needs of electricity market participants.
- Third, the robustness of different forecasting paradigms that evaluate the performance of different forecasting paradigms—classical, probabilistic, and deep learning—specifically during atypical demand conditions like public holidays or extreme weather peaks.—remains insufficiently explored, particularly from a probabilistic perspective.

Addressing these limitations requires a comprehensive framework capable of evaluating different forecasting paradigms across multiple regions, operational scenarios, and market conditions.

**Table 1:** Comparative review of electricity demand forecasting models for day-ahead and hour-ahead applications.

Ref.	Model			Data scope / region	Forecasting horizon	Key finding
	Deep learning / artificial intelligence	Statistical / linear	Stochastic / state-sspace			
^2^	✔	✖	✖	Chandigarh, India	Short-term	Direct comparative study found LSTM had “superior performance” and the lowest prediction error, 13.5% lower than SVM
^10^	✖	✔	✔	Residential and national-level demand (Saudi Arabia)	Hour-ahead & Day-ahead	SARIMA effectively captures daily and weekly seasonality for short-term horizons
^12^	✖	✔	✖	Hanoi, Vietnam	Day-ahead	Confirmed SARIMA’s robustness in emerging market data with clear seasonal cycles
^11^	✖	✔	✖	Ghana national grid	Day-ahead	Highlighted the model’s interpretability and transparency in system operation contexts
^13^	✖	✔	✖	Turkish Electricity Market	Day-ahead	Noted SARIMA’s limitations under non-linear and highly dynamic load conditions
^17^	✖	✖	✔	Electricity price series (U.S.)	Hour-ahead	Early demonstration of HMM’s strength in modeling hidden demand or price regimes
^19^	✔	✖	✔	Residential buildings (Seoul, South Korea)	Hour-ahead	MM effectively modeled uncertainty and outperformed ANN and SVM in noisy, high-frequency consumption data
^20^	✖	✖	✔	Delhi (India)	Hour-ahead	Suggested HMM as a practical probabilistic alternative when training data is limited
^22^	✔	✖	✖	Smart-meter residential data (U.S.)	Hour-ahead & Day-ahead	Established LSTM’s superior performance over traditional models for high-frequency, non-linear load data
^23^	✔	✖	✖	Four U.S. utilities	Day-ahead	Demonstrated LSTM’s scalability across multiple regional datasets
^33^	✔	✔	✖	United Arab Emirates (UAE) and Portugal	Day-ahead	Strength (LSTM): Captures non- linearity. (SARIMA): Cannot adapt well to environmental factors
^28^	✔	✖	✖	Turkey	Day-ahead	Integrating the N-HiTS forecasting model with MILP trading enables second-life EV batteries to achieve up to 85.48% of maximum arbitrage profit
^29^	✔	✖	✖	Spain	Day-ahead	The TiDE-based forecasting framework improved operational flexibility and enabled profitable energy management despite lower-than-expected PV generation
^30^	✖	✖	✖	Vietnam (VPP)	Day-ahead	A Virtual Power Plant (VPP) uses a two-stage model to manage renewable and demand uncertainty, enabling energy storage to maximize profits in real-time markets while honoring day-ahead commitments
^31^	✔	✖	✖	England	Day-ahead	ANFIS and LSTM algorithms provide the highest accuracy for short-term electricity forecasting when factoring in temperature, calendar, and pricing data
^32^	✔	✖	✖	Tokyo	Day-ahead	Regional weather variations— especially temperature and solar radiation—are essential for predicting grid imbalances because they uniquely drive both local electricity demand and renewable energy supply
This Study	✔	✔	✔	Japan (All 9 Regions)	Day-ahead Hour- ahead	- No single model is universally superior; forecasting performance is highly context and region- dependent - Improved forecasting accuracy significantly reduces daily financial burdens in the electricity market

To address these gaps, this study provides a multi-regional, scenario-based comparative analysis across all nine Japanese utility areas, while directly linking technical forecasting accuracy to its economic implications within the JEPX market framework. Specifically, this research conducts a comprehensive assessment of three distinct forecasting paradigms—SARIMA (classical statistical), HMM (probabilistic), and LSTM (deep learning)—applied across the 50/60 Hz frequency divide, nine JEPX regions under multiple forecasting horizons (day-ahead and hour-ahead) and operational “stress-test” scenarios (Day of maximum demand, Day of minimum demand, and public holiday). This multi-dimensional evaluation aims to identify which methodological framework is most suitable for Japan’s fragmented electricity market, considering both forecasting accuracy and regional adaptability. It moves beyond the generic question of “which model is best?” to the far more nuanced and practical question of “which model, under which conditions, for which region, is the most appropriate?” This moves the discussion beyond a simple model comparison toward a strategic framework for regional grid management based on localized demand behavior. This objective is achieved through the following contributions:

- Comparative analysis across methodologies: The paper conducts a systematic comparison of forecasting accuracy using three distinct modeling paradigms—SARIMA, LSTM, and HMM—addressing the gap of limited cross-paradigm evaluations in STLF.
- Evaluation across different forecasting horizons: The study examines model performance for multiple time horizons (hour-ahead and day-ahead), addressing the operational strategic divide and highlighting how forecasting accuracy and value can differ by horizon.
- Regional and contextual assessment: Forecasting models are applied across nine regions with diverse geographical and socioeconomic characteristics, filling the gap of insufficient context-aware evaluation and exploring how the same methodology performs under varying external conditions.
- Identification of key drivers: The study analyzes the factors influencing model performance in each region, helping to explain why the exact model may perform differently depending on local conditions and data characteristics.
- Recommendation of optimal approaches: Based on the comparative results and analysis of driving factors, the study identifies the best-performing forecasting approach for each region, providing actionable guidance for region-specific STLF implementation.
- Quantification of the financial consequences of forecasting errors: A key practical contribution of this study is the introduction of an economic assessment framework that monetizes forecasting inaccuracies. By translating forecasting errors (MAPE) into JPY-denominated financial burdens, the proposed approach quantifies the economic implications of forecasting model selection within the JEPX market structure.

The remainder of this paper is organized as follows. Section  “Modeling framework” outlines the modeling framework, detailing the methodology behind the SARIMA, HMM, and LSTM forecasting approaches. Section  “Model validation and scenario-based forecasting” shows the model validation and scenario-based forecasting. Section  “Results and discussion” presents the results and discussion. This section synthesizes the empirical findings from the Japan Electric Power Exchange case study, offering a comparative analysis of model performance across different regions, horizons, and demand scenarios, as well as an evaluation of the financial implications of forecast errors. Section  “Study limitations” addresses the limitations of the study, discussing constraints related to data availability and methodological scope. Section  “Conclusion” provides the concluding remarks, summarizing the key insights and policy implications, and outlines potential directions for future research.

## Modeling framework

**Fig. 1:**
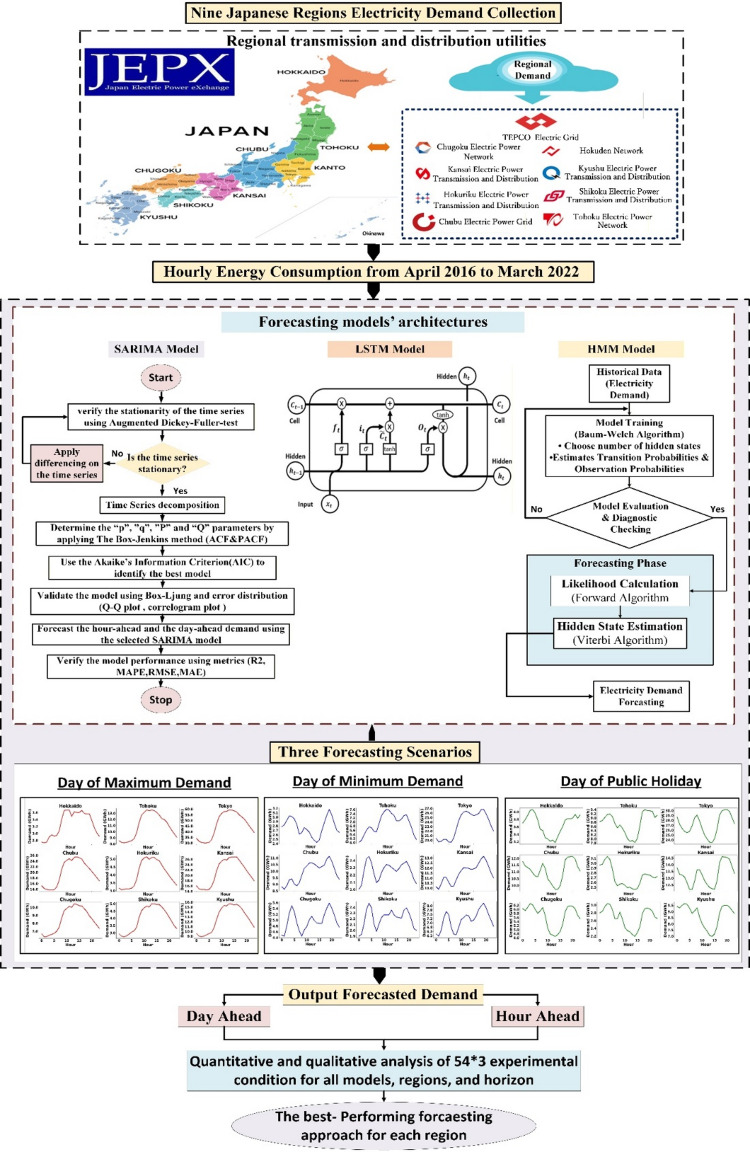
Conceptual architecture of the comparative analysis in this study.

As mentioned earlier, this study develops a comprehensive methodological framework to apply and compare three forecasting paradigms—SARIMA, HMM, and LSTM for short-term electricity demand prediction within the JEPX. The objective is to evaluate the effectiveness of each model in capturing the unique temporal dynamics, stochastic transitions, and nonlinear dependencies of electricity consumption across Japan’s nine regional power systems. Using hourly demand data from April 2016 to March 2022, the proposed framework systematically analyzes regional demand behavior under diverse operating conditions, implementing different forecasting horizons. The methodology aims to determine the best-performing approach for each region, providing valuable insights into model suitability, regional heterogeneity, and data-driven forecasting accuracy in the JEPX market environment. Figure 1 illustrates the overall methodological framework proposed in this study, which is structured into four main stages: (1) Regional data acquisition, (2) Forecasting model development, (3) Scenario-based forecasting, and (4) Model evaluation and performance analysis. At the first stage, hourly electricity demand data are collected for nine Japanese regions through the regional transmission and distribution utilities: Hokkaido, Tohoku, Tokyo, Chubu, Hokuriku, Kansai, Chugoku, Shikoku, and Kyushu. These nine regions represent Japan’s zonal electricity markets, which operate under limited inter-regional interconnections and distinct regional characteristics. Together, they form the national electricity market structure coordinated under the JEPX. The second stage develops three forecasting models, SARIMA, HMM, and LSTM, each selected to represent a distinct forecasting paradigm. The SARIMA model captures linear and seasonal temporal dependencies through differencing and parameter estimation based on the Box–Jenkins methodology. The HMM employs a probabilistic framework to model stochastic transitions in demand states using the Baum–Welch and Viterbi algorithms. Meanwhile, the LSTM network learns long-term nonlinear dependencies through recurrent gating mechanisms, enabling it to model complex temporal correlations in the load data. In the third stage, three representative forecasting scenarios are examined, including the day of maximum demand, the day of minimum demand, and a public holiday, each capturing distinct consumption behaviors and operational contexts. These scenarios facilitate the evaluation of forecasting model performance under diverse load conditions, ranging from extreme peaks to low-demand periods and socially influenced fluctuations. The contrasting demand profiles across these scenarios highlight the heterogeneity in consumption behavior. Each forecasting model is subsequently trained and validated for both day-ahead and hour-ahead horizons to rigorously assess its robustness across diverse temporal, spatial, and behavioral settings. The final stage performs a quantitative and qualitative comparative analysis across all nine regions, three models, and three scenarios, amounting to 54 × 3 experimental conditions. Model performance is assessed to determine the most accurate forecasting method for each region.

### Regional data acquisition

Japan’s electricity market serves as a compelling case study for STLF due to its unique combination of structural fragmentation and profound regional heterogeneity. This inherent complexity creates a challenging environment where a one-size-fits-all forecasting approach is insufficient, making it an ideal case study for comparing the performance of diverse modeling techniques. The market’s complexity manifests in two primary ways.

**Fig. 2:**
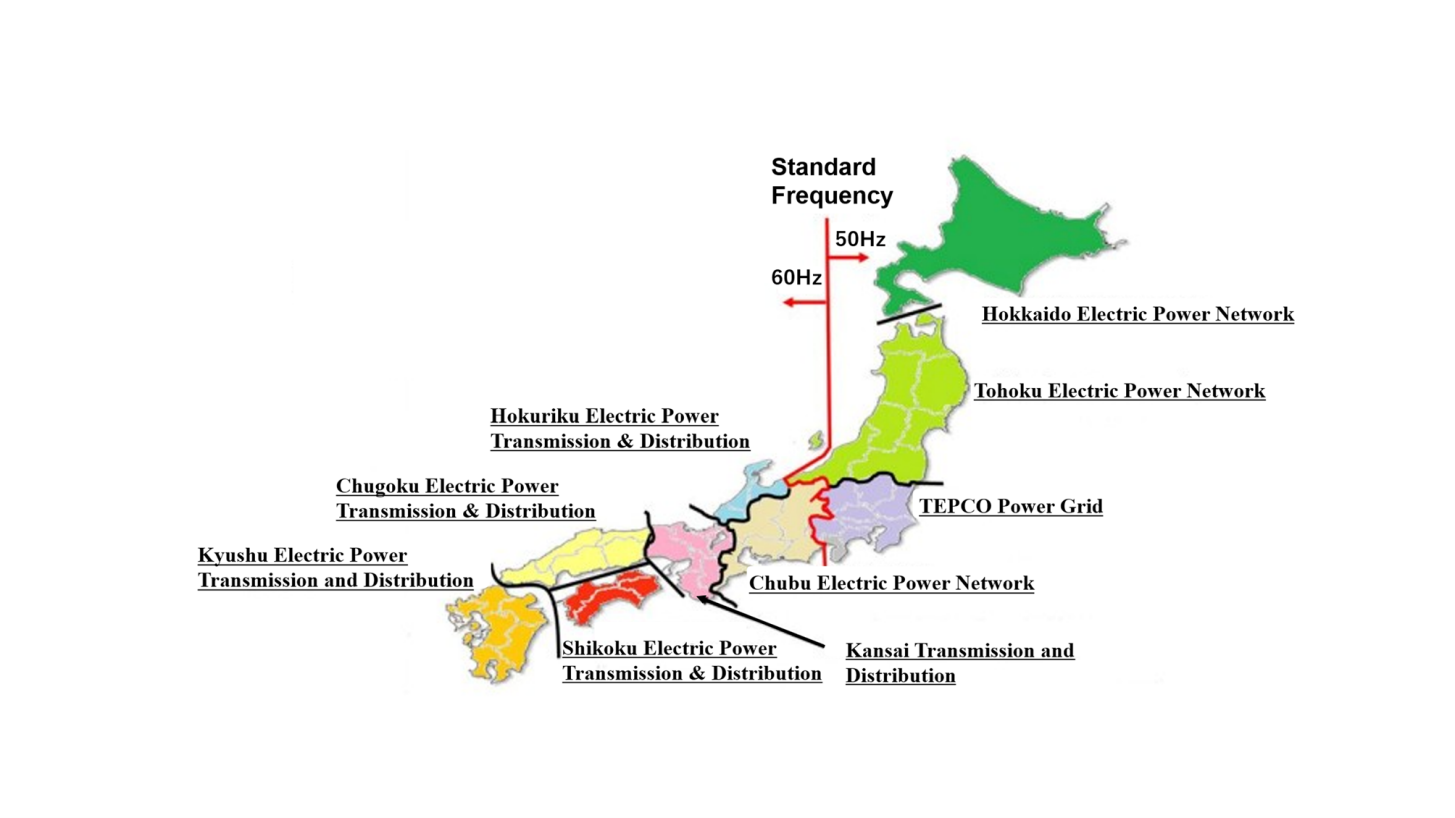
Study areas: geographic locations of the nine regions in Japan^38^.

1. Structural Fragmentation: A historical anomaly has left Japan’s power grid split into two asynchronous frequency zones: a 50 Hz network in Eastern Japan and a 60 Hz network in Western Japan. The limited capacity of the high-voltage direct current (HVDC) converters linking these zones creates a significant bottleneck, restricting the free flow of power across the country^34^. Furthermore, the grid is subdivided into nine primary service areas, as shown in Fig. 2, which are connected by interconnection lines of finite capacity. This physical structure frequently leads to transmission congestion, which effectively segments the JEPX and causes electricity spot prices to diverge, sometimes significantly, between regions.
2. Regional demand diversity: Layered on top of this structural fragmentation is a deep-rooted diversity in the electricity demand characteristics of each of the nine regions. These distinct regional profiles are shaped by a unique combination of local factors^35^:
  Climate: The Japanese archipelago exhibits a stark climatic gradient. Northern regions like Hokkaido and Tohoku have high winter demand driven by heating needs, whereas warmer southern regions like Kyushu and Shikoku experience sharp demand spikes in the summer due to air conditioning loads.
  Socioeconomic structure: The socioeconomic structure of each region significantly impacts its load profile. The Chubu region, for instance, has a robust industrial and manufacturing sector, which results in high and relatively stable demand. In contrast, the Tokyo region experiences significant commercial and residential demand^36^.
  Population and urbanization: The density and distribution of the population shape consumption patterns. Highly urbanized regions like Kansai (Osaka, Kobe, Kyoto) have different demand rhythms than more rural regions^37^.

This profound heterogeneity, summarized in Table 2, underscores that successful market participation requires a granular understanding and prediction of the unique supply-demand dynamics within each region. This makes a region-by-region comparative analysis of forecasting models not merely an academic exercise, but a direct response to the central strategic challenge facing actors in the Japanese electricity market.

**Fig. 3:**
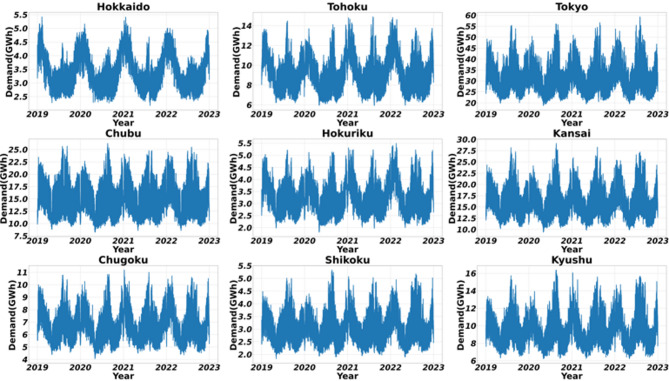
Cumulative load trend across the nine regions of Japan, based on hourly demand data from January 2019 to December 2022.

This study uses historical hourly load data for all nine major electricity regions in Japan, collected from the respective regional Transmission System Operators (TSOs)^39–47^. The dataset spans a four-year period from January 1, 2019, to December 31, 2022. This high-resolution, multi-year timeframe provides a robust foundation for training and validating the forecasting models, ensuring they are exposed to a wide range of seasonal patterns, economic conditions, and significant load-shaping events. Figure 3 illustrates the cumulative demand trend across the nine regions during this period. Table 3 summarizes the statistical properties of the hourly load demand for the nine Japanese regions (2019–2022), providing a rigorous mathematical foundation for the selected forecasting paradigms. The Mean and Standard Deviation illustrate the significant scale disparity across the Japanese grid, with the Tokyo region (32,307.3 MW) representing a load intensity nearly ten times that of smaller regions like Shikoku (3,096.6 MW). To evaluate relative volatility independent of scale, the Coefficient of Variation (CV) was calculated; the high CV values in Tokyo (20.79%) and Chubu (20.63%) indicate a high degree of demand fluctuation, justifying the use of complex non-linear models like LSTM to capture these dynamics. Furthermore, the distribution shape analysis reveals consistent Positive Skewness across all regions (ranging from 0.433 to 0.790), signifying a “heavy-tail” distribution where demand frequently peaks above the mean—a critical factor for market clearing price volatility. The Kurtosis values range from platykurtic (-0.749 in Hokkaido) to leptokurtic (0.436 in Kyushu), highlighting regional differences in peak concentration that necessitate a diversified modeling approach. Finally, the Augmented Dickey-Fuller (ADF) test results yield highly negative statistics and $\:p\:$values of 0.000 for all regions, empirically confirming the stationarity of the processed time-series and validating the mathematical stability of the SARIMA and HMM frameworks used in this study.

**Table 2:** Characteristics of Japan’s nine major electric power regions^37^.

Region	Incumbent utility	Frequency	Generation capacity (MW)	Population (M)	Major cities/load centers	Key demand drivers
Hokkaido	Hokkaido Electric Power	50 Hz	8,300	5.2	Sapporo, Hakodate	Significant winter heating demand; moderate industry
Tohoku	Tohoku Electric Power	50 Hz	16,800	11.2	Sendai, Niigata	High winter heating demand; agriculture and industry
Tokyo	Tokyo Electric Power	50 Hz	66,800	43.8	Tokyo, Yokohama, Kawasaki	Massive commercial and residential hub; summer cooling
Chubu	Chubu Electric Power	60 Hz	33,400	17.1	Nagoya, Shizuoka	Heavy industry (automotive); summer cooling
Hokuriku	Hokuriku Electric Power	60 Hz	8,500	3.0	Kanazawa, Toyama	Industrial base; significant winter snowfall impacting demand
Kansai	Kansai Electric Power	60 Hz	34,260	20.7	Osaka, Kobe, Kyoto	Major commercial, industrial, and residential center
Chugoku	Chugoku Electric Power	60 Hz	11,530	7.4	Hiroshima, Okayama	Industrial and manufacturing base
Shikoku	Shikoku Electric Power	60 Hz	5,770	3.8	Matsuyama, Kochi	Mixed residential and industrial; summer cooling
Kyushu	Kyushu Electric Power	60 Hz	29,820	13	Fukuoka, Kumamoto	High solar PV penetration; significant summer cooling demand

**Table 3:** Statistical properties of load demand.

Region	Mean (MW)	Median (MW)	Std_Dev	CV_%	Skewness	Kurtosis	ADF_Statistic	p_value
Hokkaido	3481.28	3382	632.87	18.18	0.43	-0.75	-5.77	< 0.05
Tohoku	9376.07	9280	1663.91	17.75	0.46	-0.34	-8.77	< 0.05
Tokyo	32307.26	31,700	6716.79	20.79	0.60	0.02	-11.06	< 0.05
Chubu	15152.31	14,843	3125.99	20.63	0.48	-0.24	-15.02	< 0.05
Hokuriku	3318.79	3266	618.07	18.62	0.46	-0.21	-11.97	< 0.05
Kansai	16269.45	15875.5	3203.75	19.69	0.68	0.13	-11.25	< 0.05
Chugoku	6803.88	6655	1155.75	16.99	0.58	0.00	-11.00	< 0.05
Shikoku	3096.64	3030	563.15	18.19	0.69	0.33	-12.11	< 0.05
Kyushu	9674.72	9377	1696.49	17.54	0.79	0.44	-10.91	< 0.05

**Fig. 4:**
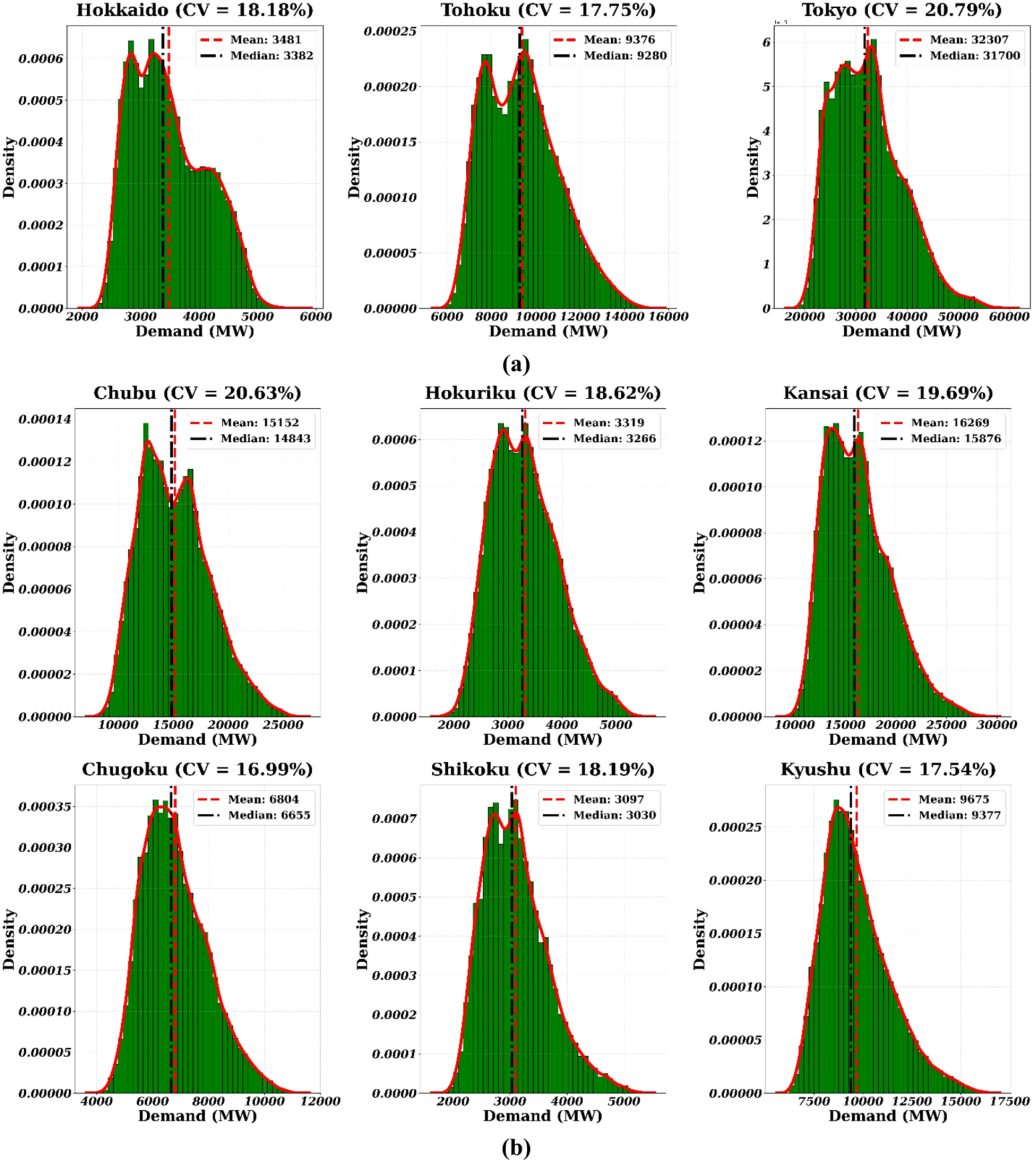
KDE distributions and CV for electricity consumption across the nine regions of Japan, based on hourly demand data from January 2019 to December 2022.a) East Japan b)West Japan.

To further illustrate regional demand heterogeneity, Fig. 4 represents the Kernel Density Estimation (KDE) distributions of electricity consumption and the calculated CV for each of the nine regions. This visualization provides a clear comparative snapshot of the distinct demand characteristics where CV, defined as the ratio of the standard deviation to the mean of the hourly load, normalizes demand fluctuations across regions with different average consumption levels, a high CV indicates a more volatile and less predictable demand pattern, posing a greater forecasting challenge, while a low CV reflects more stable and consistent consumption. The plots reveal significant differences in the shape, spread, and central tendency of the load distributions, quantitatively confirming the heterogeneity of Japan’s regional markets and highlighting the diverse challenges that forecasting models must address in each unique operational context.

### Forecasting model development

To ensure robustness and interpretability, three forecasting models were developed, each representing a distinct methodological paradigm: statistical, probabilistic, and deep learning.

- **SARIMA Model – Statistical Approach**

The SARIMA model extends the conventional ARIMA framework by incorporating seasonal components to account for periodic patterns in time-series data. It is specified as $\:SARIMA\left(p,d,q\right)\left(P,D,Q\right)s$, comprising non-seasonal $\:\left(p,d,q\right)$ and seasonal $\:\left(P,D,Q\right)$ parameters, where $\:s$ represents the seasonal cycle length. In this study, $\:s$ = 24 reflects the hourly seasonality inherent in daily electricity consumption patterns. The SARIMA formulation integrates autoregressive (AR), integrated (I), and moving average (MA) operators across both seasonal and non-seasonal dimensions, allowing it to represent linear dependencies, trends, and cyclical behaviour simultaneously. The residual component is assumed to follow a white noise process with constant variance and zero mean, ensuring unbiased forecast estimation. As illustrated in Fig. 1, the SARIMA implementation adheres to the Box–Jenkins methodology. The process begins with stationarity testing using the Augmented Dickey–Fuller (ADF) test, followed by appropriate differencing to eliminate non-stationarity in both seasonal and non-seasonal components. Model identification is guided by Autocorrelation (ACF) and Partial Autocorrelation (PACF) plots to specify candidate orders for AR and MA terms. Model estimation and selection are performed, using the Akaike Information Criterion (AIC) to balance goodness of fit with parsimony. Diagnostic checking ensures that residuals exhibit no autocorrelation or heteroscedasticity through the Ljung–Box test and residual normality assessments as detailed in the Supplementary Material Section 1.1. Once validated, the SARIMA model is employed to generate hour-ahead and day-ahead forecasts, providing a robust benchmark for comparison with nonlinear and probabilistic approaches^11^.

(b) **Hidden Markov Model (HMM) – Probabilistic Approach**

The HMM was employed to model stochastic regime transitions in electricity consumption by representing unobserved (hidden) demand states that evolve over time. A continuous observation HMM is characterized by three key parameter sets: the initial state distribution, the state transition probability matrix, and the observation probability distribution. Together, these components define the probabilistic structure governing transitions between hidden demand states and the generation of observed electricity load values. As illustrated in Fig. 1, historical electricity demand data were used to train the HMM via the Baum–Welch algorithm, which estimates both the transition and emission probabilities. The optimal number of hidden states was determined through likelihood-based evaluation using information criteria such as the AIC and the Bayesian Information Criterion (BIC), ensuring a balance between model accuracy and parsimony. Following model training, the Forward algorithm was applied to compute observation likelihoods, while the Viterbi algorithm inferred the most probable sequence of hidden states over time, as detailed in the Supplementary Material Section 1.2^48^.

(c) **Long Short-Term Memory (LSTM) – Deep Learning Approach**

The LSTM network is a nonlinear, data-driven modeling approach designed to capture long-term temporal dependencies in electricity demand. As illustrated in Fig. 1, the LSTM architecture comprises three fundamental gates—the forget gate, input gate, and output gate—which collectively control information retention, updating, and propagation across time steps. The forget gate selectively removes irrelevant past data from the cell state, while the input gate determines which new information should be stored. The output gate then decides how much of the updated cell state is revealed as the current output, allowing the network to preserve useful temporal information while filtering noise. This mechanism enables the LSTM to efficiently capture complex and nonlinear demand patterns driven by temporal dynamics and behavioural variations. The model was trained using the Adam optimizer with the MSE as the loss function and validated through systematic train–test partitioning across regional datasets. The Adam optimizer was selected for its ability to integrate the benefits of both Root Mean Square Propagation (RMSProp) and Adaptive Gradient Algorithm (AdaGrad). This choice is specifically motivated by the inherent volatility of multi-regional load data, where the optimizer’s momentum terms facilitate smoother convergence in the presence of noisy gradients^49^. By utilizing adaptive learning rates, the framework effectively mitigates the risk of becoming trapped in local minima—a common challenge when modeling the non-linear demand surges of large-scale grids like Tokyo and Chubu. This design allows robust forecasting performance, particularly in modelling nonlinear interactions and dynamic responses to external and temporal factors, as detailed in the Supplementary Material Section 1.3^50^.

## Model validation and scenario-based forecasting

### Statistical model validation across seasonal test data

To ensure the reliability and generalization capability of the forecasting models, a comprehensive validation framework was implemented using historical electricity demand data from the nine Japanese regional electricity systems covering the period 2019–2022. The dataset consists of hourly load demand observations, providing a sufficiently large time series for robust model training and evaluation.

The dataset was divided into training and testing subsets using an 80%–20% split, where 80% of the data was used for model training, and the remaining 20% were reserved for out-of-sample validation. This approach allows the predictive performance of the SARIMA, HMM, and LSTM models to be evaluated on unseen data, ensuring that the results reflect the models’ generalization ability rather than overfitting to historical observations.

Table 4 presents the performance evaluation of the forecasting models using widely adopted statistical metrics, including MAPE, RMSE, and Mean Absolute Error (MAE). These indicators provide complementary measures of forecasting accuracy by capturing relative error, squared deviations, and absolute differences between the predicted and observed electricity demand values.

This validation procedure ensures that the forecasting models are evaluated using a statistically rigorous framework based on the full historical dataset, thereby confirming their predictive reliability before conducting scenario-based analysis.

### Scenario-based forecasting

In addition to statistical validation across the full test dataset, a scenario-based analysis was conducted to illustrate model performance under representative operational conditions.

Three representative demand scenarios were selected:

1. Maximum demand scenario – representing extreme peak consumption conditions observed on August 2, 2022, typically associated with high temperatures and intensive cooling demand.
2. Minimum demand scenario – representing low electricity consumption periods illustrated by May 5, 2022, this date occurs during Japan’s Golden Week national holiday, when many industrial and commercial activities are temporarily suspended, resulting in a noticeable reduction in electricity demand across the power system.
3. Public holiday scenario – representing irregular consumption patterns influenced by social behavior, analyzed using January 1, 2023 (New Year’s Day), a national holiday characterized by a noticeable reduction in electricity demand.

The maximum and minimum demand days were identified from the historical demand dataset as the days with the highest and lowest total electricity demand, respectively. These representative days were selected to analyze the system performance under extreme demand conditions. These scenarios provide insight into how forecasting models respond to extreme, low-demand, and irregular consumption patterns, which are important for electricity market operations.

For each scenario, both day-ahead and hour-ahead forecasting horizons were analyzed across the nine regional electricity markets in Japan. In total, the experimental framework includes 162 forecasting evaluations (9 regions × 3 models × 2 horizons × 3 scenarios), enabling a comprehensive comparison of model performance under different operational contexts.

By combining statistical validation using the full test dataset with scenario-based analysis, the proposed framework provides a robust evaluation of forecasting model performance across Japan’s fragmented electricity market.

**Table 4:** Model performance across the test data.

Model	SARIMA			LSTM			HMM		
Region	MAPE	RMSE	MAE	MAPE	RMSE	MAE	MAPE	RMSE	MAE
Hokkaido	1.70%	70.26	55.03	1.13%	48.30	36.65	1.50%	57.53	47.84
Tohoku	1.37%	172.68	123.93	0.81%	98.96	73.38	1.48%	160.26	130.81
Tokyo	1.28%	575.10	408.20	0.76%	345.66	244.59	1.82%	764.74	573.21
Chubu	1.16%	233.67	165.82	0.74%	142.54	107.96	1.95%	374.51	282.02
Hokuriku	1.51%	65.70	48.15	0.86%	35.77	27.10	1.88%	72.20	56.46
Kansai	1.33%	211.86	173.23	0.80%	168.83	126.10	1.77%	367.07	282.08
Chugoku	1.82%	160.55	119.71	1.20%	102.91	78.03	1.63%	129.78	104.22
Shikoku	1.92%	79.44	58.86	1.17%	48.66	35.56	1.85%	74.26	56.67
Kyushu	1.09%	144.76	103.27	0.93%	118.79	87.87	1.77%	230.41	168.39

## Results and discussion

This section presents the empirical results of the multi-faceted comparative analysis of SARIMA, LSTM, and HMM for STLF across Japan’s nine electricity regions. The evaluation is designed to analyze model performance across different scenarios and timeframes, explore the reasons behind regional performance differences, and culminate in a set of actionable, region-specific forecasting strategies.

### Comparative forecasting performance: a scenario-based evaluation

To understand the specific strengths and limitations of each modeling paradigm, this section provides a granular analysis of the three distinct demand scenarios. By integrating qualitative observations from the hourly forecast plots with quantitative performance metrics, a nuanced picture of model behaviour emerges. To facilitate a rigorous quantitative comparison, the MAPE, RMSE, and MAE values for all models, regions, scenarios, and horizons are consolidated into two primary tables. Table 5 presents the performance for the strategic Day-Ahead horizon, while Table 6 details the performance for the operational Hour-Ahead horizon. The detailed setup for each model, including architectural specifications and parameter tuning, is provided in the supplementary material section 2.

**Table 5:** Estimated values of statistical indicators on the day-ahead for all three models across each region and scenario.

Scenarios	Max demand day			Min demand day			Public holiday		
Region	SARIMA	LSTM	HMM	SARIMA	LSTM	HMM	SARIMA	LSTM	HMM
MAPE									
Hokkaido	3.63%	2.30%	17.90%	5.62%	17.18%	12.33%	4.10%	5.03%	11.57%
Tohoku	3.30%	1.64%	8.56%	3.30%	2.66%	20.15%	6.93%	14.40%	4.03%
Tokyo	6.31%	4.55%	14.43%	3.40%	1.84%	42.95%	16.44%	12.86%	13.27%
Chubu	4.36%	2.28%	18.17%	9.38%	1.59%	10.41%	23.69%	5.98%	9.56%
Hokuriku	4.77%	1.22%	6.76%	5.07%	8.99%	10.90%	14.04%	15.13%	20.22%
Kansai	2.52%	2.30%	15.06%	3.48%	2.84%	13.31%	17.27%	6.63%	6.51%
Chugoku	2.72%	2.80%	9.68%	3.73%	2.12%	31.30%	9.62%	15.1%	10.11%
Shikoku	4.37%	1.35%	11.85%	7.43%	8.17%	18.86%	7.10%	11.58%	14.17%
Kyushu	3.40%	2.58%	8.25%	1.81%	3.31%	24.03%	19.93%	7.1%	5.69%
RMSE									
Hokkaido	160. 3	86.0	585.2	188.0	522.3	442.0	182.5	217.6	465.1
Tohoku	414.37	217.2	1146.9	292.3	213.9	1465.1	683.6	1494.9	412.4
Tokyo	3936.6	2491.3	9532.3	903.9	505.7	11354.8	4662.6	4081.6	4769.9
Chubu	1109.2	533.2	4721.3	1061.5	198.9	1258.3	2839.4	760.4	1195.9
Hokuriku	211.4	56.1	403.2	129.1	228.2	298.1	395.0	438.1	593.9
Kansai	657	702.2	4205.6	524.7	406.4	2228.4	2546.2	1010.7	1149.7
Chugoku	243.96	260.4	1141.4	232.0	123.0	1854.0	627.5	933.7	711.1
Shikoku	182.5	57.8	594.3	190.3	184.6	469.3	231.0	336.9	399.0
Kyushu	530.3	416.0	1380.9	161.5	289.4	2542.9	1855.4	646.6	550.1
MAE									
Hokkaido	125.45	75.8	564.5	148.9	462.8	347.5	150.5	181.0	405.3
Tohoku	366.2	174.0	978.6	228.3	184.6	1365.0	586.7	1212.3	351.8
Tokyo	3218.8	2183.3	7568.9	779.2	432.0	9380.0	4324.2	3383.2	3398.0
Chubu	942.3	468.3	3907.0	951.2	157.8	1026.0	2569.4	645.9	1026.2
Hokuriku	174	48.9	308.7	116.6	203.6	247.6	368.9	397.2	527.2
Kansai	553.4	530.6	3503.8	399.2	324.7	1562.2	2270.8	885.2	848.5
Chugoku	224.89	240.4	909.2	190.9	106.0	1570.1	503.7	809.8	521.9
Shikoku	168.2	48.8	503.1	168.3	179.2	416.1	173.0	294.9	361.6
Kyushu	459	353.4	1144.7	127.4	226.2	1766.6	1653.1	598.9	481.7

**Table 6:** Estimated values of statistical indicators on the hour-ahead for all three models across each region and scenario.

Scenarios	Max Demand day			Min demand day			Public holiday		
Region	SARIMA	LSTM	HMM	SARIMA	LSTM	HMM	SARIMA	LSTM	HMM
MAPE									
Hokkaido	1.38%	0.91%	1.64%	2.09%	1.43%	2.15%	1.89%	2.03%	1.35%
Tohoku	1.12%	0.84%	1.32%	1.32%	1.22%	2.97%	1.32%	1.40%	1.45%
Tokyo	0.83%	0.63%	4.9%	1.15%	0.75%	4.17%	1.29%	0.91%	1.55%
Chubu	0.53%	0.41%	3.6%	1.22%	0.63%	6.89%	1.62%	1.18%	2.89%
Hokuriku	1.06%	0.67%	1.89%	1.17%	0.94%	6.26%	1.68%	1.24%	2.9%
Kansai	0.48%	0.60%	2.45%	0.93%	0.76%	3.06%	1.33%	1.19%	1.59%
Chugoku	0.94%	0.98%	1.78%	1.90%	1.26%	3.54%	1.71%	1.15%	2.18%
Shikoku	1.22%	0.67%	2.67%	2.37%	1.57%	4.53%	1.94%	2.25%	1.87%
Kyushu	0.87%	0.74%	3.97%	1.24%	1.34%	5.02%	1.62%	1.03%	1.53%
RMSE									
Hokkaido	53.3	36.0	61.3	69.2	49.2	67.0	80.0	90.9	59.3
Tohoku	151.3	124.5	177.3	125.8	104.0	271.0	141.5	142.1	153.1
Tokyo	468.9	421.9	3589.1	322.6	218.5	1095.2	516.5	326.6	508.8
Chubu	145.7	108.5	1075.1	182.3	73.2	808.9	231.4	161.3	372.3
Hokuriku	48.8	36.6	100.6	31.9	26.0	172.4	55.2	42.8	85.7
Kansai	143.5	171.0	740.9	138.0	114.7	475.6	211.9	184.1	239.6
Chugoku	95.4	101.4	177.7	115.0	89.8	222.9	127.0	88.7	144.6
Shikoku	60.7	31.7	137.4	64.0	43.6	128.7	64.8	76.6	56.1
Kyushu	143.9	117.4	714.2	116.2	119.9	455.6	177.9	110.0	156.8
MAE									
Hokkaido	45.0	28.9	52.7	57.3	38.9	58.5	67.9	72.3	49.5
Tohoku	126.4	94.7	146.6	92.5	84.4	193.3	114.5	124.1	127.8
Tokyo	353.3	280.0	2542.2	267.4	171.2	897.7	359.4	247.0	415.1
Chubu	106.1	82.6	800.9	122.2	61.4	650.4	175.5	129.2	314.6
Hokuriku	41.4	27.0	78.3	26.0	21.3	135.4	45.5	33.7	76.8
Kansai	98.2	129.5	554.7	108.1	88.2	326.4	173.2	157.1	212.0
Chugoku	79.2	84.2	149.6	94.3	61.3	169.2	95.8	65.2	117.5
Shikoku	47.0	26.4	111.5	52.1	34.5	94.2	51.0	59.5	48.6
Kyushu	110.2	97.4	557.0	88.3	95.5	337.9	138.9	89.3	131.1

A qualitative review of the hourly forecast plots provides an intuitive understanding of each model’s behaviour across the diverse scenarios, as follows.

#### Scenario 1 (maximum demand day): forecasting under high-stress conditions

**Fig. 5:**
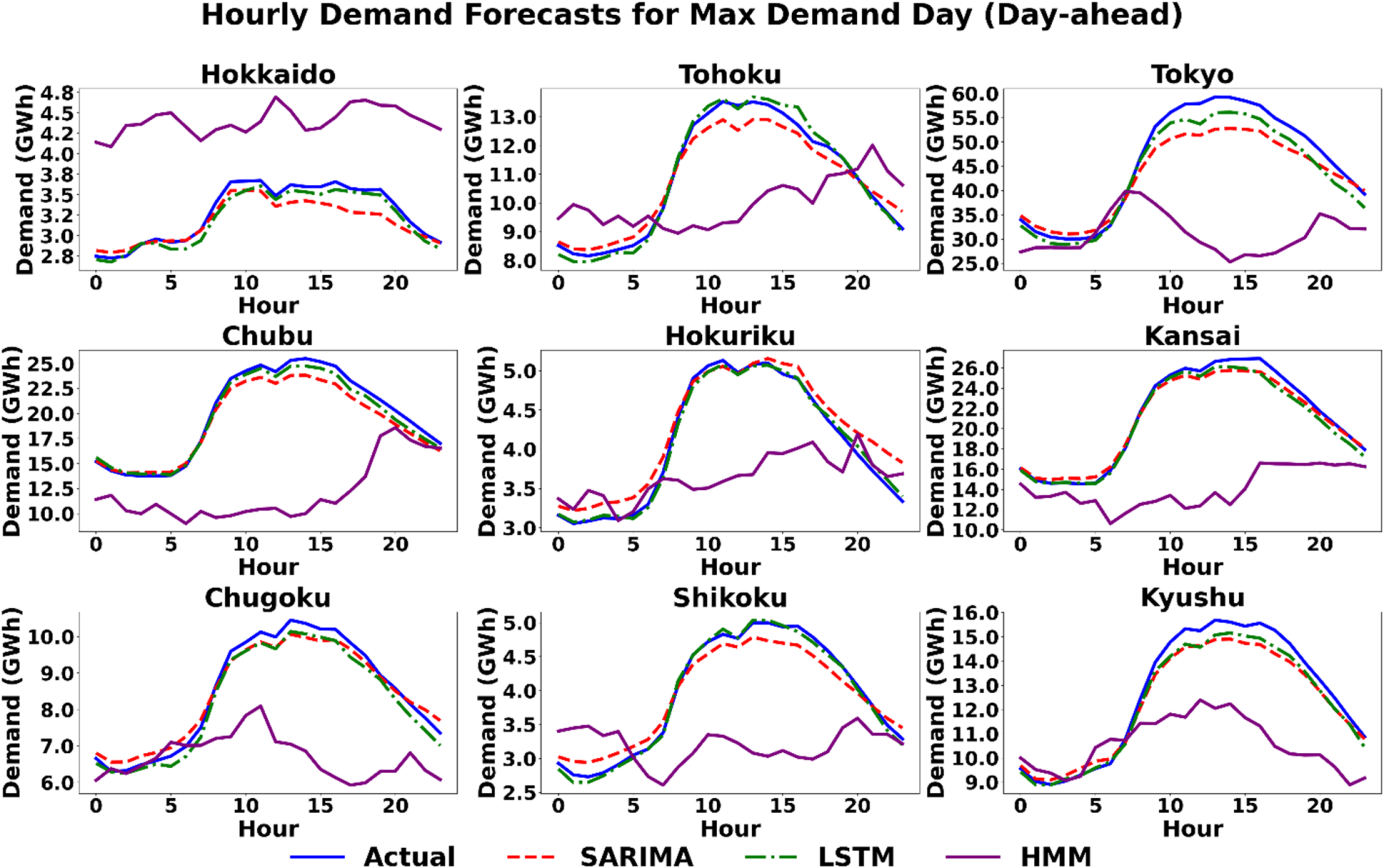
Scenario 1: Hourly demand forecasting on the day of maximum demand (Day-Ahead) across nine regions using the three models.

**Fig. 6:**
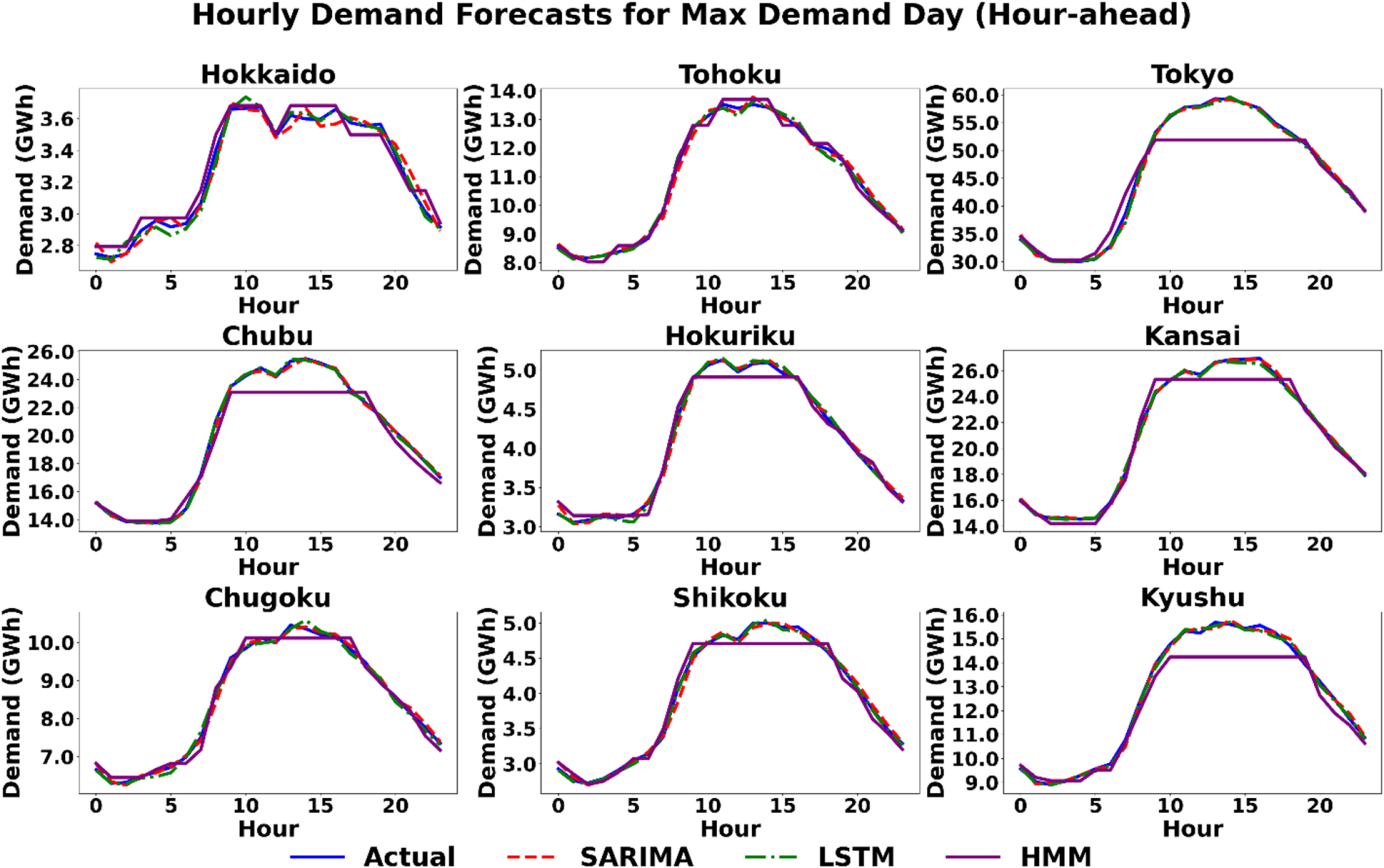
Scenario 1: Hourly demand forecasting on the day of maximum demand (Hour-Ahead) across nine regions using the three models.

The maximum demand day scenario serves as a critical stress test, evaluating each model’s capacity to accurately predict the pronounced diurnal load cycle that pushes the power grid to its operational limits. A qualitative review of the hourly forecast profiles in Figs. 5 and 6 provides deeper context for this scenario, which is characterized by a strong and predictable diurnal cycle. Both the SARIMA and LSTM models demonstrate a strong ability to capture the fundamental shape of the demand curve, closely tracking the actual load. In contrast, the HMM model consistently and significantly underestimates the magnitude of the load across nearly all regions. For the hour-ahead horizon shown in Fig. 6, the SARIMA and LSTM models track the actual demand with remarkable precision. The HMM model, however, exhibits a distinct behaviour, producing a “flat-topped” forecast during peak hours in all regions except (e.g., Hokkaido, Tohoku), indicating an inability to model the nuanced peak of the demand curve at this shorter horizon. The quantitative results in Tables 5 and 6 reflect this visual assessment. For day-ahead forecasts, both SARIMA and LSTM models exhibit strong performance. Although their MAPE values are often comparable, the LSTM model shows a lower error in seven of the nine regions. The inclusion of RMSE and MAE metrics further distinguishes the models, particularly in high-load regions. For example, in Tokyo, the LSTM achieves an RMSE of 2491.3 MW compared to SARIMA’s 3936.6 MW, indicating that while percentage errors may seem close, the LSTM significantly reduces large-magnitude deviations. In some cases, the performance is very close, such as in Kansai (2.52% for SARIMA vs. 2.30% for LSTM) and Chugoku (2.72% for SARIMA vs. 2.80% for LSTM). However, there are regions where the performance difference is more pronounced, such as in Hokuriku, where the LSTM achieves a MAPE of 1.22% compared to SARIMA’s 4.77%. In contrast, the HMM consistently underperforms in this scenario, as reflected in its high MAPE values, such as 17.90% in Hokkaido and 18.17% in Chubu. In the hour-ahead horizon, accuracy improves significantly for all models, marked by a drastic reduction in RMSE and MAE values across the board. The LSTM model is the top performer in seven regions, while SARIMA achieves the lowest error in the remaining two (Kansai and Chugoku), with MAPE values falling below 1% for both models in these instances. The HMM model struggles significantly at this shorter horizon. While its MAPE is competitive in Tohoku (1.32%), it is substantially higher than the other models in all other regions, reaching 4.9% in Tokyo. This poor quantitative performance is explained by its “flat-topped” forecast shape during peak hours in several major regions, as shown in Fig. 6, indicating an inability to model the nuanced peak of the demand curve.

#### Scenario 2 (minimum demand day): modeling low-demand dynamics

**Fig. 7:**
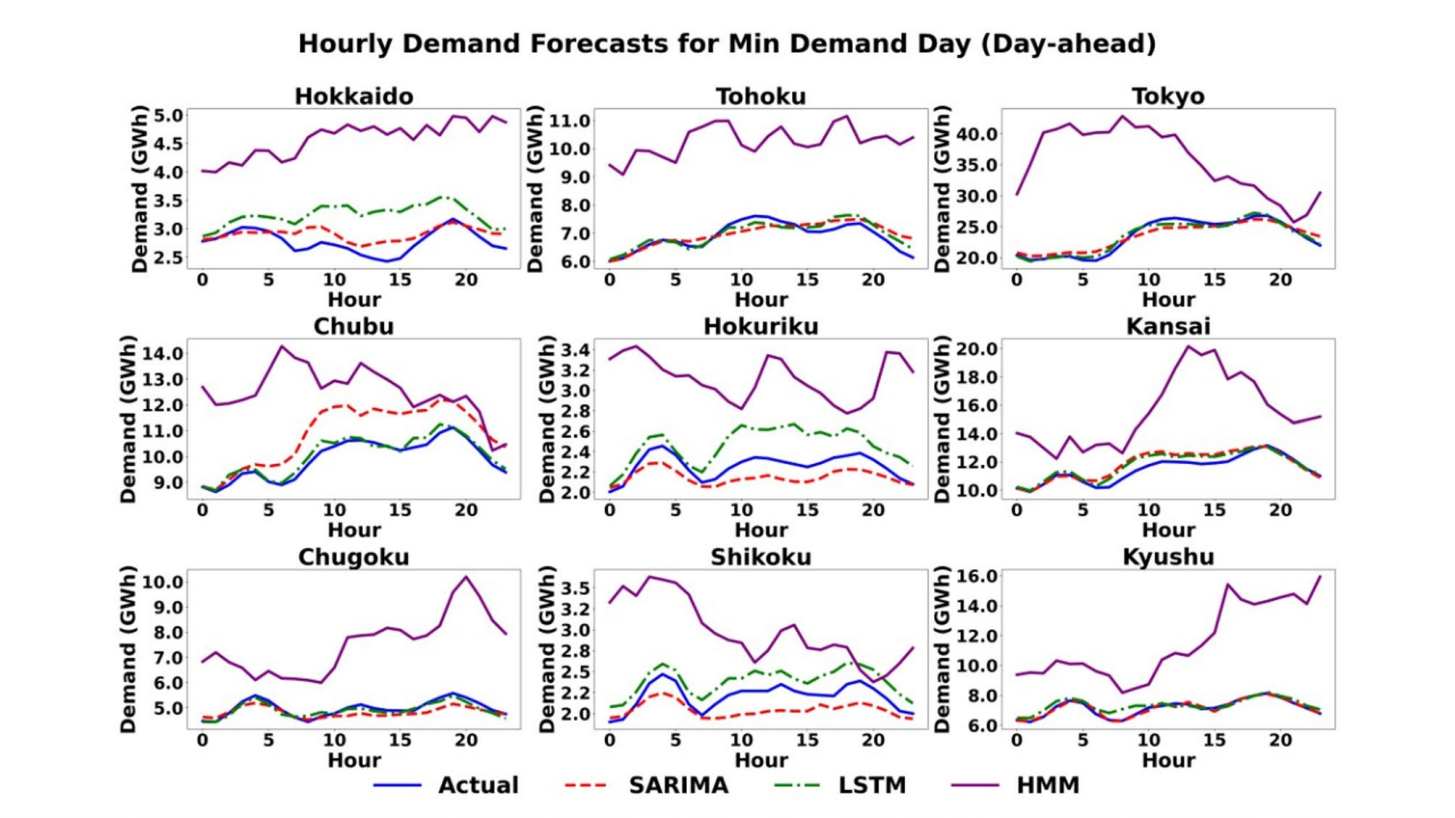
Scenario 2: Hourly demand forecasting on the day of minimum demand (Day-Ahead) across nine regions using the three models.

**Fig. 8:**
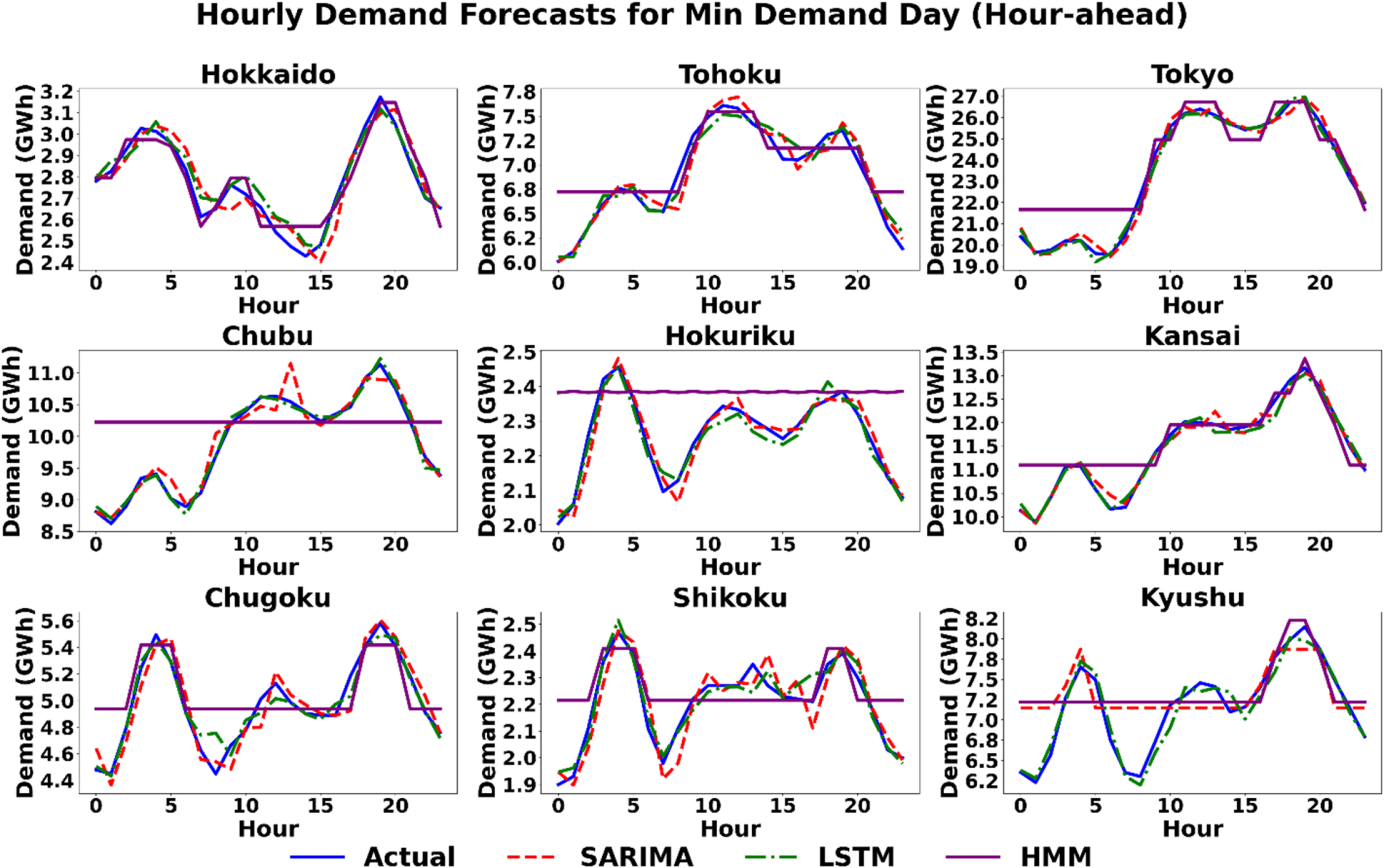
Scenario 2: Hourly demand forecasting on the day of minimum demand (Hour-Ahead) across nine regions using the three models.

This scenario assesses model performance under conditions of less pronounced diurnal patterns, which often occur during shoulder seasons or weekends. Qualitatively, the day-ahead plots in Fig. 7 show a challenging context where both SARIMA and LSTM tend to over-forecast in a few regions, while the HMM model exhibits a pattern of significant over-forecasting across all regions. In the hour-ahead horizon shown in Fig. 8, all models struggle to capture the high-frequency volatility of the actual demand. The SARIMA and LSTM models provide smoothed approximations, while the HMM produces a distinct, jagged, step-like forecast that often fails to match the actual demand’s peaks and troughs. Quantitatively, the day-ahead results in Table 5 show a varied performance landscape. The LSTM model is the top performer in five regions, including the major load centres of Tokyo (1.84%) and Chubu (1.59%), while SARIMA performs best in the other four, including an exceptionally lower MAPE, such as in Hokkaido (5.62% for SARIMA vs. 17.18% for LSTM and 12.33% for HMM) and Kyushu (1.81% for SARIMA compared to 3.31% for LSTM and 24.03% for HMM). This is corroborated by the absolute error metrics; for instance, in Chubu, the LSTM records an RMSE of 198.9 MW, significantly outperforming SARIMA (1061.5 MW) and HMM (1258.3 MW), highlighting its precision even during lower demand periods. The pronounced performance degradation of the HMM during Tokyo’s minimum demand scenarios highlights the limitations of state-space models in capturing the multi-scalar temporal dependencies of a massive urban load center. Unlike the LSTM, which effectively manages the ‘memory’ of past demand cycles, the HMM’s reliance on immediate state-transition probabilities results in a ‘lag effect’ during the atypical load troughs characteristic of off-peak periods. For the hour-ahead horizon in Table 6, the benefit of informational proximity is clear. The LSTM model registers a lower MAPE in eight of the nine regions, though the SARIMA model remains highly competitive, with the performance difference often being less than half a percentage point.

#### Scenario 3 (Public Holiday): the challenge of atypicality

**Fig. 9:**
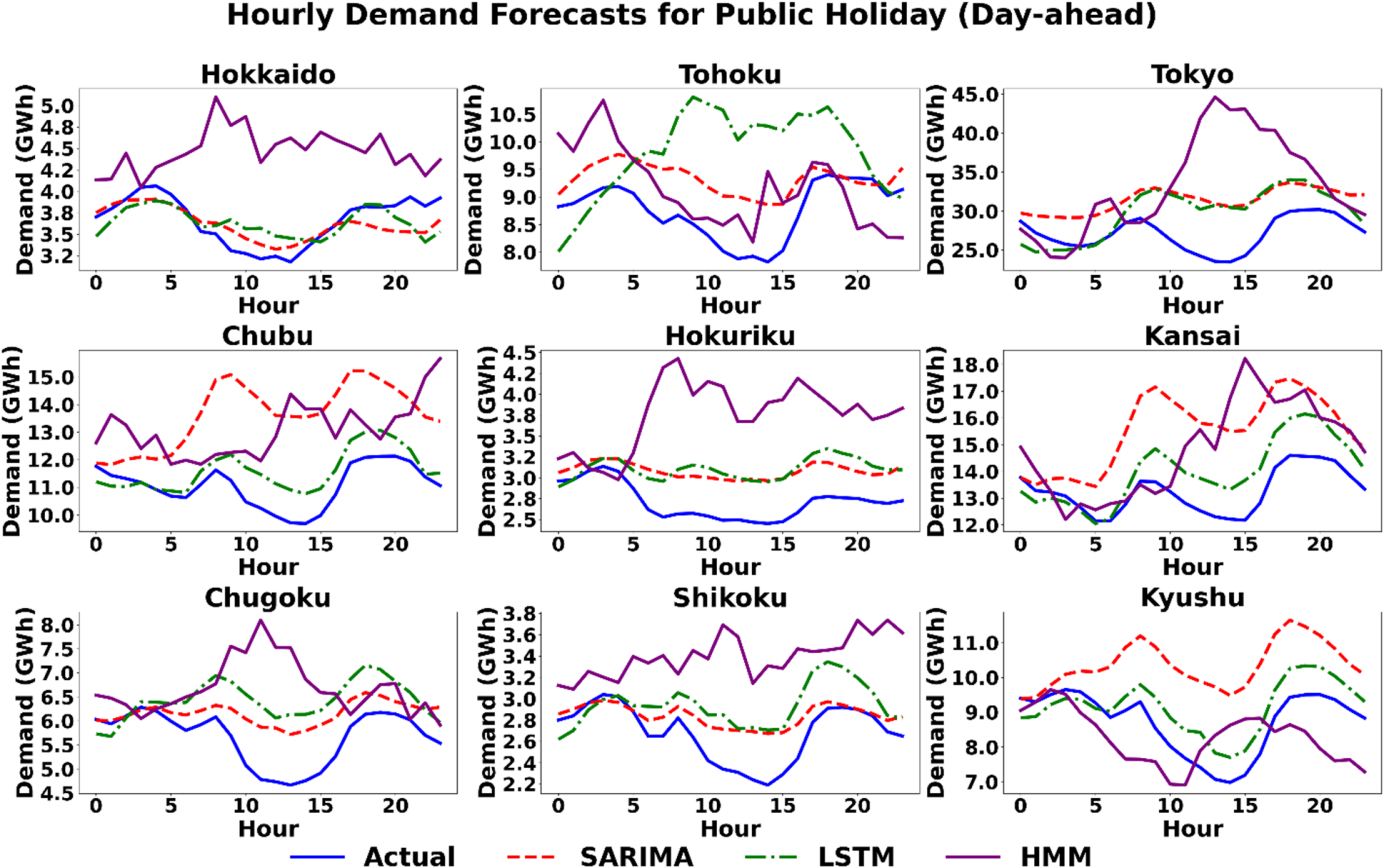
Scenario 3: Hourly demand forecasting on the day of the Public Holiday (Day-Ahead) across nine regions using the three models.

This scenario represents the most rigorous test of model adaptability, as it introduces a structural break from typical weekly patterns. The Day-ahead hourly forecast plots in Fig. 9 visually confirm the challenges faced by all the models. The results from this scenario disrupt the normal performance hierarchy and reveal the distinct strengths of each modeling paradigm. SARIMA, which relies on fixed seasonality parameters derived from historical weekly patterns, faces significant challenges. Although it remains the optimal gay-ahead model in four regions (Hokkaido, Hokuriku, Chugoku, and Shikoku), its error rates are generally higher than in other scenarios, reflecting its difficulty in adapting to the atypical load shape. Conversely, the results reported in Table 5 reveal that this scenario highlights the unique capabilities of the HMM. Its performance improves dramatically, making it the optimal day-ahead model in three regions: Tohoku, Kansai, and Kyushu. In Tohoku, the HMM’s advantage is evident across all metrics, achieving an RMSE of 412.4 MW and MAE of 351.8 MW, which are substantially lower than SARIMA’s RMSE of 683.6 MW and MAE of 586.7 MW. This paradoxical strength in the most unpredictable scenario can be attributed to its architecture. A public holiday represents a clear shift from a “weekday state” to a “holiday state,” and the HMM’s structure is uniquely suited to capture this abrupt, state-based change. The LSTM model proves its value by delivering the lowest error in the two largest and most complex regions, Tokyo and Chubu, demonstrating its ability to learn and generalize from a variety of complex patterns, including non-standard days.

**Fig. 10:**
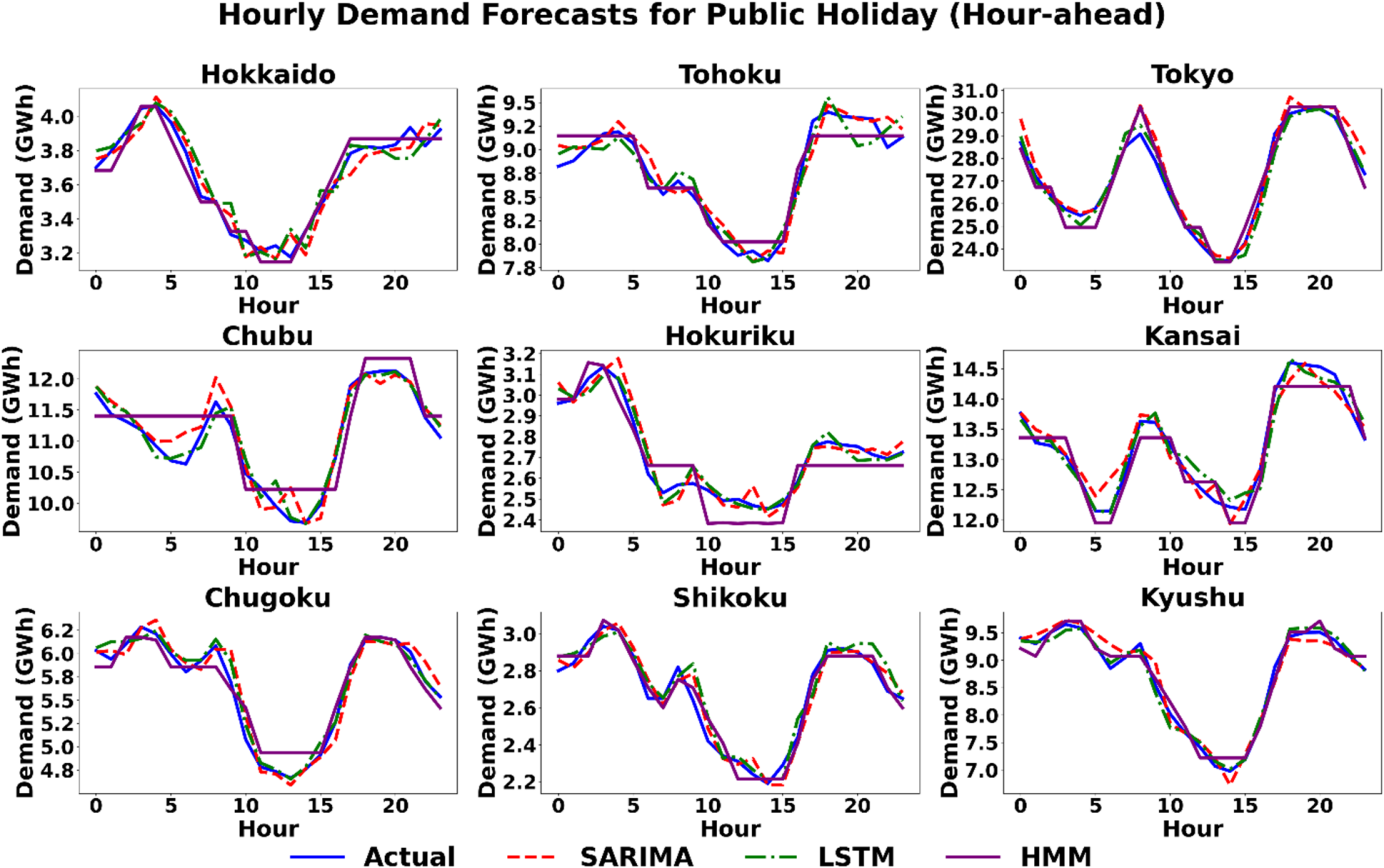
Scenario 3: Hourly demand forecasting on the day of the Public Holiday (Hour-Ahead) across nine regions using the three models.

In the hour-ahead horizon shown in Fig. 10, the visual difference is striking; all three models demonstrate a remarkable ability to track the actual demand curve very closely, showcasing the powerful corrective effect of recent data. Even models that produced poor day-ahead forecasts converge toward the actual load, with their predictions becoming nearly indistinguishable from one another. The LSTM model emerges as the top performer in six regions. However, the performance across all three models is remarkably comparable. SARIMA achieves the lowest error in Tohoku (1.32%), while the HMM model is optimal in Hokkaido (1.35%) and Shikoku (1.87%). The narrow margins in many regions highlight that all models become highly competitive at this shorter horizon, even on an atypical day. This demonstrates the HMM’s strength in adapting to non-standard demand patterns, making it a viable contender, especially when compared to its weaker performance on more regular days.

### Optimal model selection: a visual and analytical synthesis

**Fig. 11:**
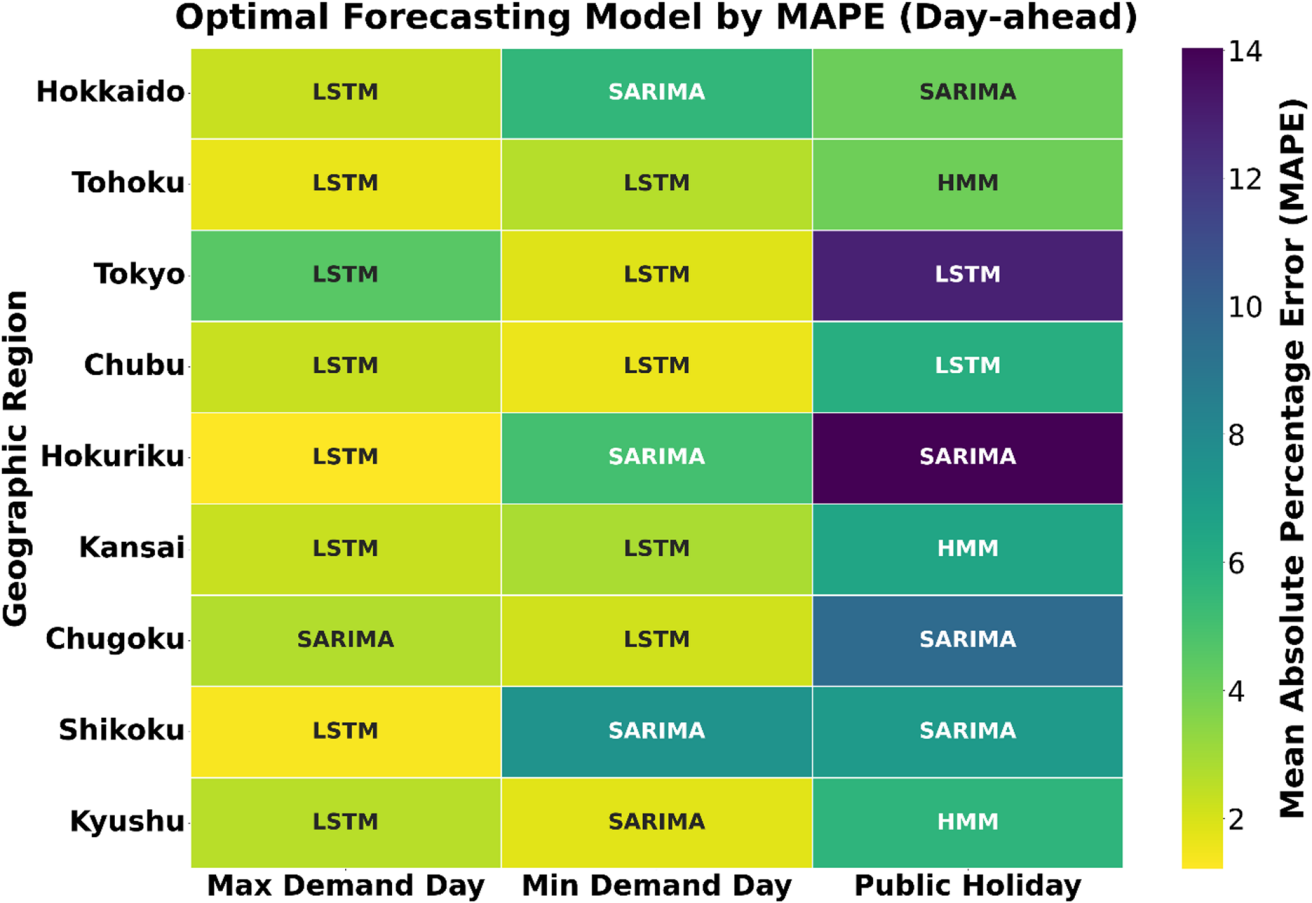
Optimal forecasting model by MAPE (Day-ahead) through the nine regions.

**Fig. 12:**
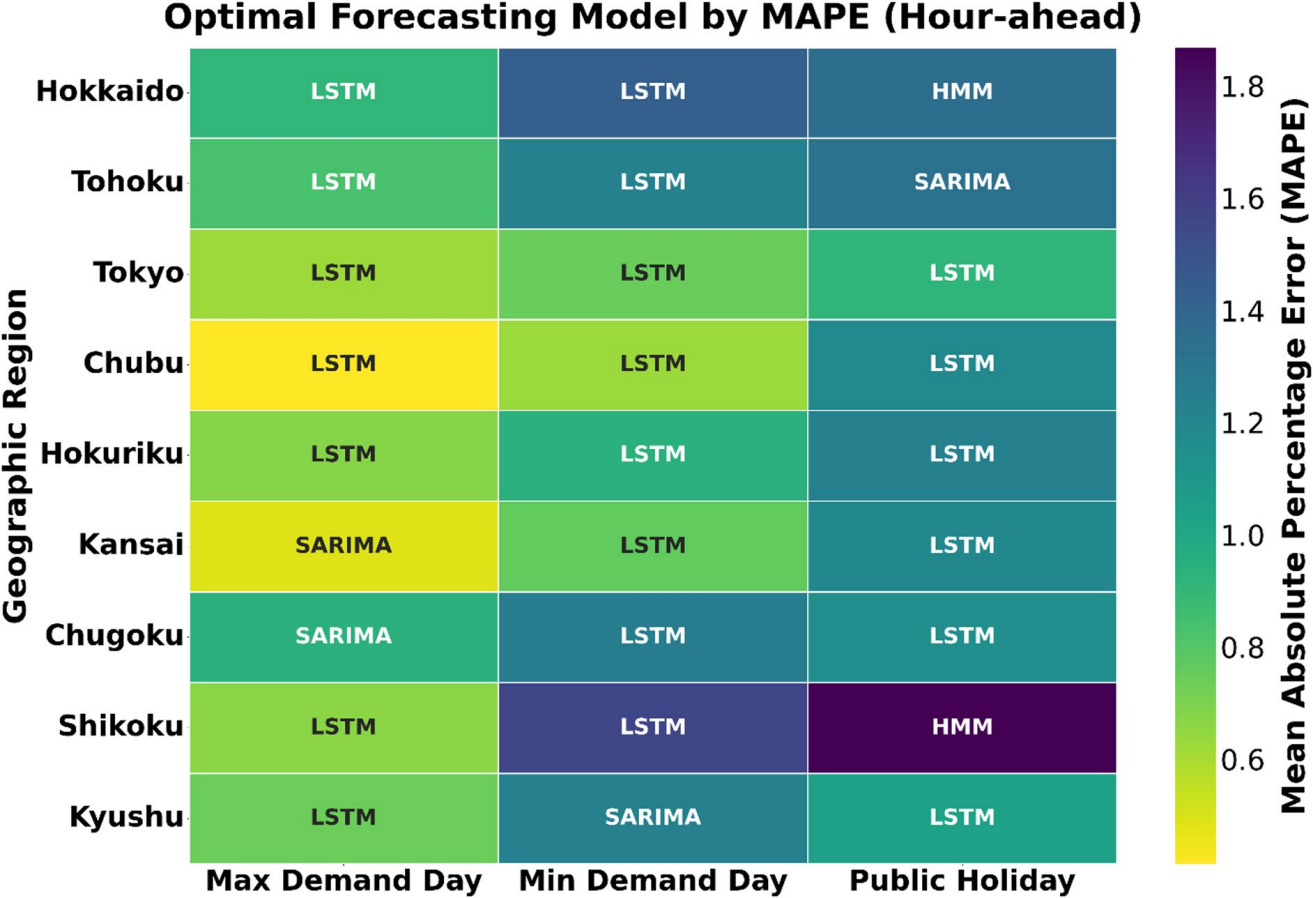
Optimal forecasting model by MAPE (Hour-ahead) through the nine regions.

To synthesize the detailed results from the performance tables into an intuitive visual summary, this section leverages a series of purpose-built visualizations. Tables 5 and 6 provide a side-by-side comparison of the MAPE for all three models across every region, scenario, and forecasting horizon. This detailed view is then consolidated in Figs. 11 and 12, which serve as visual heatmaps indicating the single optimal model for each of the 54 experimental conditions, offering an immediate, high-level perspective on model suitability under varying contexts. This section synthesizes the findings to unpack the underlying drivers of performance variation and provides a framework for context-aware model selection.

The Day-ahead summary in Fig. 11 reveals a complex patchwork, clearly demonstrating that no single model is universally dominant for strategic forecasting. It shows a strong LSTM presence in the high-stakes Maximum Demand scenario, reflecting its strength in handling complex temporal dynamics. However, this dominance is not absolute; in regions like Chugoku and Kansai, SARIMA performs competitively, sometimes even surpassing LSTM, such as in Chugoku. The heatmaps also reveal a mixed SARIMA/LSTM pattern under the Minimum Demand scenario and a highly fragmented landscape during the Public Holiday, where all three models find specialized niches. This illustrates that context-aware model selection is crucial, as model performance varies with both regional characteristics and scenario-specific complexities. The hour-ahead summary in Fig. 12 presents a markedly different landscape where LSTM emerges as the top performer in the majority of cases.

#### LSTM’s superiority in high-volatility urban centers

**Fig. 13:**
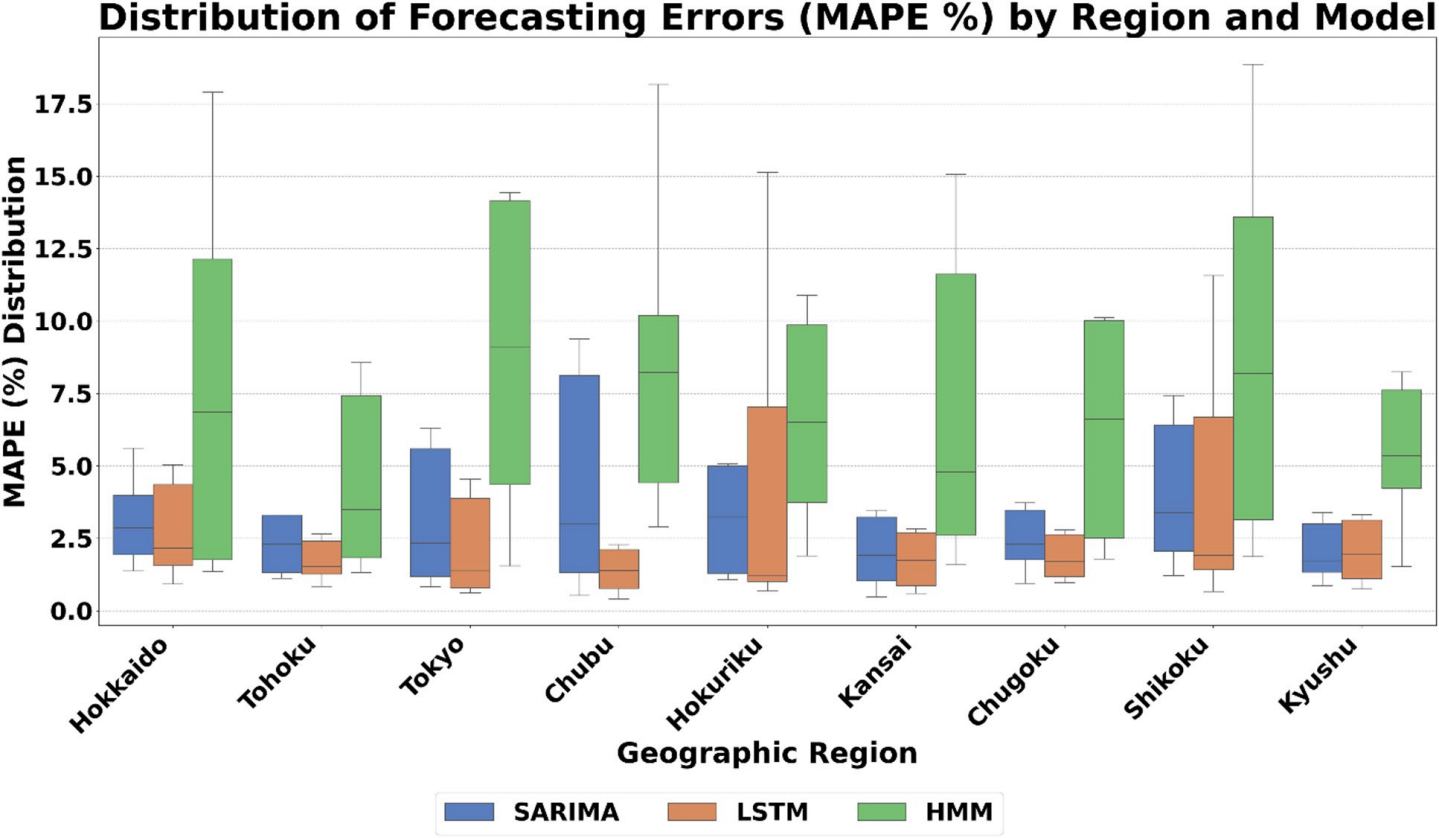
The distribution of forecasting errors through the nine regions.

The LSTM network’s ability to learn complex, non-linear temporal dependencies allows it to excel in high-density, commercially driven regions such as Tokyo, Chubu, and Kansai. These regions exhibit highly dynamic demand profiles due to the interaction of commercial, industrial, and residential activities. As shown in Fig. 13, LSTM achieves the lowest median MAPE and exhibits the tightest error distributions in these areas. This supports the principle of complexity matching, that the internal complexity of the model must align with the structural complexity of the regional demand signal. Linear models like SARIMA often fail to capture such non-linearities, especially during atypical or high-volatility periods such as public holidays.

#### SARIMA’s niche in stable, industrially-driven regions

Despite LSTM’s dominance in many hour-ahead contexts, SARIMA remains a strong secondary candidate in regions characterized by more stable, industrially driven demand. Such regions as Chugoku, Kyushu, and Hokuriku, with large concentrations of steel, petrochemical, shipbuilding, and automotive industries, show SARIMA’s relative advantage. Its parsimonious and transparent structure efficiently captures strong, regular seasonal patterns, making it ideal where the load signal is periodic and predictable. For example, its 1.81% day-ahead MAPE in Kyushu on the minimum demand day represents the best performance recorded in that entire scenario. In such settings, the simplicity of SARIMA prevents overfitting, allowing it to outperform deep learning models whose flexibility may cause them to track minor fluctuations (noise) rather than the dominant periodic structure.

#### HMM’s better performance in specific operational contexts, particularly in public holiday scenarios

HMM’s strength lies in capturing discrete regime shifts and abrupt state transitions. In regions such as Hokkaido and Shikoku, where grid behavior exhibits discontinuities during holidays, HMM identifies these hidden operational states with notable accuracy. The visual and analytical synthesis across Figs. 11, 12 and 13 confirms that model optimality is inherently context-dependent. LSTM dominates where demand dynamics are complex and rapidly evolving; SARIMA excels in stable, regular systems; and HMM provides resilience in irregular, state-dependent regimes. Together, these results reinforce the importance of aligning model complexity with data complexity, forming the basis for a context-aware adaptive model selection framework in electricity demand forecasting across Japan’s regional grids.

### Uncertainty quantification and regional archetypes

While deterministic point forecasts provide a baseline for system planning, they do not fully capture the operational risks inherent in Japan’s fragmented electricity market. To move beyond deterministic metrics, forecast uncertainty can be characterized using prediction intervals (PI) derived from the statistical distribution of forecast errors^51^. Accordingly, this study incorporates UQ to define the probabilistic “risk zones” associated with each forecasting paradigm. The forecast residuals are approximated using a Gaussian probability density function centered at zero mean ($\:\mu\:=0)$, while the RMSE is used as an estimate of the standard deviation $\:\sigma\:\:$to represent the dispersion of forecasting errors. Based on this formulation, a 95% PI is constructed, providing grid operators and market participants with an interpretable risk range for day-ahead electricity demand forecasting and procurement decisions.

To ensure analytical rigor, the UQ framework is applied to four representative archetypes—Tokyo, Chubu, Kansai, and Kyushu—which were selected to encapsulate the structural heterogeneities of the Japanese market, including the 50/60 Hz frequency divide, high industrial load density, and significant renewable energy penetration. By mapping these uncertainty profiles, the study demonstrates that models with narrower distributions provide higher operational certainty, whereas the wider spreads quantify the increased volatility risks inherent in specific regional grid architectures.

**Fig. 14:**
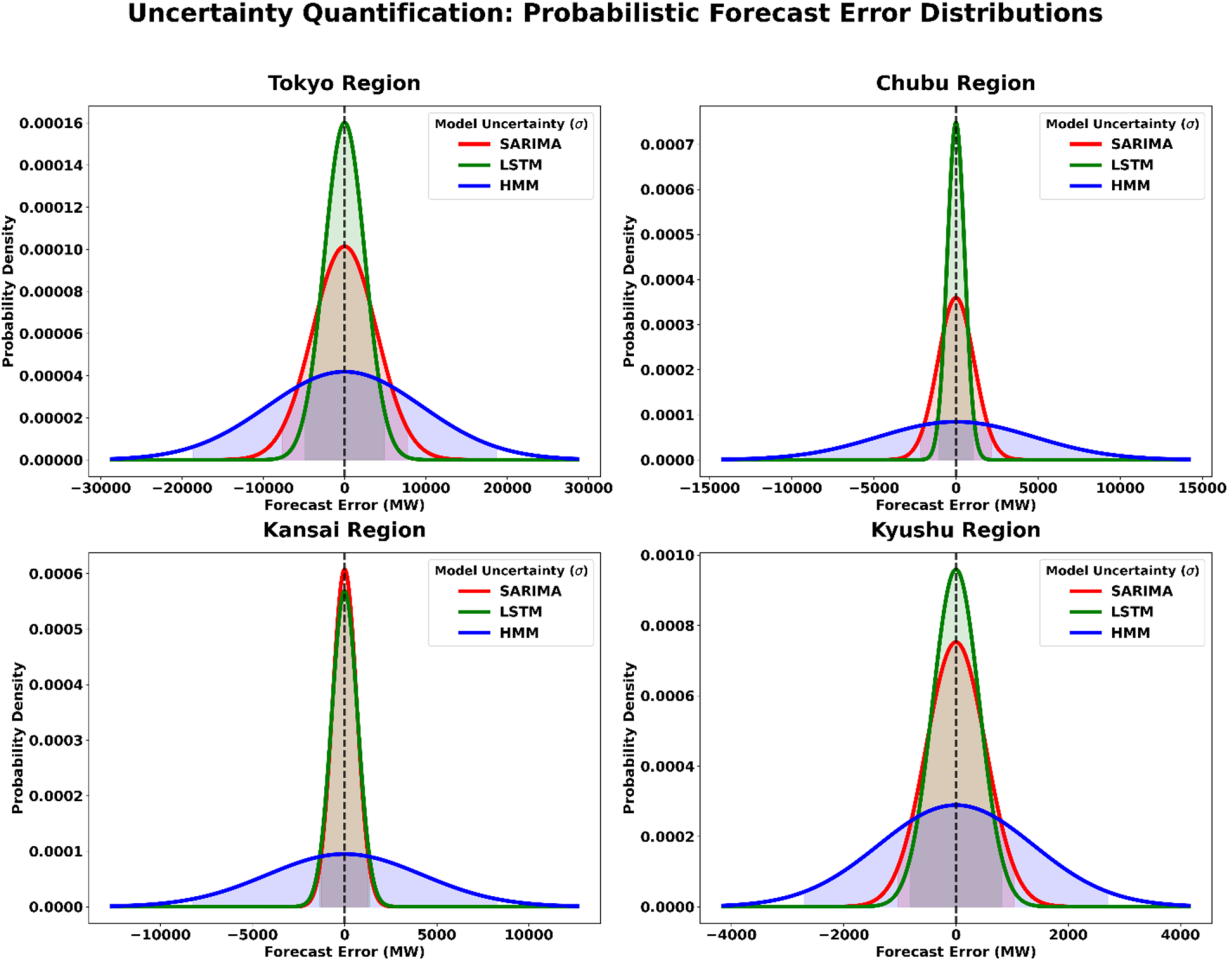
Probabilistic forecasting error distributions.

The UQ results illustrated in Fig. 14 highlight the relationship between forecasting accuracy and operational risk across the four major regions. The LSTM model consistently exhibits the narrowest and highest probability density distribution, indicating lower residual variance and greater forecasting reliability. In contrast, the HMM model shows significantly wider distributions, particularly in the Tokyo and Chubu regions, reflecting higher uncertainty and potential for large-scale imbalance associated with state-space transitions in these industrial hubs. The SARIMA model generally shows intermediate dispersion, suggesting moderate uncertainty relative to the other approaches. These probabilistic distributions provide a more informative assessment of forecasting performance by quantifying regional risk levels and enabling market participants to estimate appropriate procurement margins and reserve requirements beyond traditional deterministic error metrics.

### The financial burden of the load forecast inaccuracy in wholesale power markets

In a deregulated market like the JEPX, supply-demand match is vital, and utilities and retailers adjust the bulk of their electricity transactions in a day-ahead market based on load forecasts. Inaccurate load forecasts create supply-demand imbalances in the market and pose significant financial risks due to margin erosion for retailers. Overestimating demand results in a shortage when demand outpaces supply, which must be covered by purchasing electricity at often much higher and more volatile prices on the spot or intraday markets. Conversely, under-forecasting demand creates an oversupply, which leads to excess inventory, which forces retailers to adopt aggressive pricing strategies to sell surplus stock. This price volatility can be extreme; while average spot prices in recent years have hovered around 11–15 JPY/kWh, periods of supply tightness have seen prices surge to over 50 JPY/kWh and, in extreme cases, even exceed 200 JPY/kWh^52^. In such an environment, even minor improvements in forecasting precision can translate into substantial financial gains. The selection of an advanced forecasting model, therefore, has far-reaching economic implications for both energy procurement and reserve capacity scheduling. To quantify the direct financial impact of choosing the optimal forecasting model, this analysis considers three distinct case studies from the day-ahead results. These scenarios were selected to illustrate how even slight differences in MAPE can lead to substantial financial burden, depending on the region’s demand scale and the specific context:

- Chugoku Region (Maximum Demand Day): This case represents a scenario with a minimal performance difference. The optimal SARIMA model (2.72% MAPE) was only marginally better than the sub-optimal LSTM model (2.80%), an “error difference” of just 0.08%.
- Tokyo Region (Maximum Demand Day): This case highlights a moderate performance gap in a high-demand area. The optimal LSTM model (4.55% MAPE) significantly outperformed the sub-optimal SARIMA model (6.31%), resulting in an error difference of 1.76%.
- Tohoku Region (Public Holiday): This case demonstrates the value of model adaptability. The optimal HMM (4.03% MAPE) was substantially more accurate than the sub-optimal SARIMA model (6.93%), yielding an error difference of 2.9% on an atypical day.

To quantify the economic significance of forecasting model selection, the daily financial burden was estimated using an economic impact assessment framework introduced in this study. This framework converts forecasting errors into economic terms by first calculating the difference in MAPE between two models for each scenario and then translating this difference into an equivalent volume of misallocated energy. The resulting energy volume is subsequently monetized using the corresponding regional JEPX system price. By linking forecasting accuracy with electricity demand and market prices, this approach enables utilities to interpret abstract statistical errors in terms of practical financial impacts and operational decision-making.

Using the actual regional demand and the average JEPX system price^48^ for each specific day, the financial burden due to the oversupply or shortage in the market resulting from selecting the incorrect forecasting model is detailed in Table 7.

**Table 7:** Quantified financial burden due to forecasting error.

Region	Scenario	Actual demand (MWh/day)	Error difference^*^	Demand mismatch due to the forecasting error (MWh/day)	Average market price (Yen/kWh)	Daily financial burden (Million yen)
Chugoku	Max. demand day	203,128	0.08%	162.5	33.19	5.4
Tokyo	Max. demand day	1,098,990	1.76%	19,342.2	33.19	642
Tohoku	Public holiday	210,124	2.90%	6,093.6	16.82	102

*Error difference indicates the difference in the value of MAPE in the first and second optimal models for the selected region.

The results presented in Table 7 are noteworthy. In the Chugoku region, a seemingly minor improvement of 0.08% in MAPE translates to a potential daily financial burden of 5.4 million yen. This illustrates that even small gains in accuracy can have significant monetary implications. The situation is even more pronounced in the Tokyo region, where a larger error difference of 1.76%, combined with a high daily demand, leads to an astonishing financial burden of 642 million yen in just one day during extreme peak demand. Lastly, the Tohoku case study highlights the importance of selecting context-specific models; by choosing the appropriate model (HMM) for an unusual holiday, the team avoided an additional 2.9% error, resulting in savings of approximately 102.5 million yen.

This analysis reframes the importance of proper model selection and implementation. The costs associated with developing and deploying more advanced or context-specific models should not be viewed as an expense, but as a high-return investment in financial risk mitigation. For large-scale urban systems like Tokyo, the business case for adopting superior forecasting technology is exceptionally compelling, as the savings from even a single high-stakes day can be immense. Beyond direct financial costs, improved forecasting accuracy yields systemic benefits that enhance grid stability, facilitate decarbonization, and improve overall operational efficiency.

## Study limitations

While this study provides a rigorous comparison, several key limitations in data and methodology should be noted.

The net load data used does not account for unmeasured “invisible” generation from behind-the-meter (BTM) solar, which can bias model accuracy. The models do not explicitly incorporate new, atypical demand patterns, most notably from electric vehicle (EV) charging, which follows different patterns than traditional weather-based loads. The study relied on deterministic point forecasts (a single value), which do not capture the full range of possible outcomes. This omits a critical component for risk management, as probabilistic load forecasts (PLFs) are necessary to quantify uncertainty and allow market participants to assess the financial risk of different demand scenarios. The models were trained on a fixed historical window that includes significant structural breaks (e.g., the COVID-19 pandemic, 2022 market rule changes), which can bias model parameters and reduce forecast accuracy.

## Conclusion

This study was motivated by the critical need for accurate and reliable STLF within Japan’s unique energy landscape, which is characterized by structural fragmentation and profound regional demand heterogeneity. Recognizing that a “one-size-fits-all” approach is insufficient, this paper undertook a comprehensive comparative analysis of three distinct modeling paradigms: the classical statistical SARIMA model, the machine learning-based LSTM network, and the probabilistic HMM. The methodological framework was designed for rigor and practical relevance, evaluating these models across Japan’s nine electricity regions, two distinct forecasting horizons (Day-Ahead and Hour-Ahead), and three representative demand scenarios designed to test performance under varying conditions of stress and atypicality.

The empirical results yield several key conclusions that advance the understanding of STLF model performance. The primary finding provides critical evidence for Japanese policy-makers and utilities to move away from centralized, one-size-fits-all forecasting models. Model performance is contingent on a confluence of factors, including regional demand characteristics, the specific demand scenario, and the forecasting horizon. The question is not which model is best, but which model is best for a given context? A clear performance dynamic emerges between the SARIMA and LSTM models. LSTM generally excels in complex, non-linear environments, such as high-demand days in dense urban centers, where its ability to capture intricate patterns provides a distinct advantage. However, SARIMA demonstrates robust and often superior performance in more stable, periodic environments, where its parsimonious structure prevents overfitting and provides a more reliable forecast. Its strong comparative efficiency, even in complex scenarios, establishes it as a powerful and indispensable tool, not merely a simple baseline. The HMM, while generally underperforming in standard forecasting tasks, revealed its unique value as a specialist tool for handling structural breaks. Its ability to model discrete regime shifts makes it exceptionally well-suited for forecasting atypical events like public holidays, where it significantly outperformed both SARIMA and LSTM in a few regions.

At the operational Hour-Ahead horizon, the performance of all three models converged significantly. This highlights the overriding importance of recent data for real-time forecasting, where the immediate past provides a signal strong enough to mitigate the inherent structural differences between the models. Investment in advanced and context-appropriate forecasting models is a high-return strategy for financial risk mitigation. For large-scale, demand-dense regions like Tokyo, the economic rationale for adopting superior forecasting technology is unequivocal, as even minor improvements in accuracy mitigate substantial financial exposure in the JEPX Day-Ahead and Spot markets. These insights carry broader policy implications for the JEPX operator. First, they highlight the need for market rules that explicitly incentivize improved forecasting accuracy—such as performance-based settlement adjustments or reduced imbalance penalties for entities that deploy validated advanced models. Second, the results support the introduction of standardized forecasting quality benchmarks to reduce systemic forecasting risk across retailers. Third, enhancing data transparency and providing higher-frequency, high-resolution market and weather data would enable market participants to utilize more sophisticated forecasting architectures, thereby strengthening overall market efficiency. Finally, JEPX could consider facilitating a forecasting innovation framework—through sandbox environments, benchmarking platforms, or data-sharing consortia—to promote the adoption of context-aware models tailored to the diverse regional characteristics of Japan’s electricity system.Together, these findings demonstrate that accurate forecasting is not merely a methodological advancement but a strategic enabler of financial stability, operational resilience, and improved market design within Japan’s evolving energy landscape.

Beyond statistical evaluation, the results provide several practical implications for grid operators and market participants. First, the 95% PI derived from the multi-regional UQ framework supports optimized spinning reserve procurement, enabling Transmission System Operators to reduce unnecessary reserve allocation and associated operational costs. Second, the economic impact assessment framework provides a useful tool for risk management and strategic bidding, enabling retailers and VPPs to better estimate financial exposure in the JEPX Day-Ahead market. Finally, the findings highlight regional differences in forecasting uncertainty across Japan’s 50 Hz and 60 Hz grid zones, suggesting that frequency converter scheduling and interregional power flows can be better coordinated to enhance overall grid stability and resilience.

Future work will focus on expanding STLF models to include economic exogenous variables. A next-generation multivariate model should be tested to determine whether its accuracy improves by including data such as JEPX bidding volumes, real-time spot prices, coal prices, and even key financial indicators. Another promising direction involves developing regionally customized meta-learning frameworks that automatically identify the optimal model for each region and forecasting horizon. This would support scalable operational deployment and reduce reliance on manual model tuning. Additionally, evaluating forecasting performance under extreme or unprecedented events—such as heatwaves, typhoons, or supply disruptions—could provide insights into model robustness during periods of system stress. Finally, future research should consider the policy and market design implications of forecasting accuracy. This includes assessing how improved forecasting can optimize bidding strategies, reduce imbalance costs, support renewable integration, and contribute to long-term decarbonization goals. Collaborating with JEPX and regional utilities to develop standardized forecasting benchmarks and shared data platforms may further strengthen system-wide resilience and enhance the economic efficiency of Japan’s electricity markets.

## Supplementary Information

Below is the link to the electronic supplementary material.

Supplementary Material 1

## Data Availability

The datasets used and/or analysed during the current study are available from the corresponding author on reasonable request.

## References

[1] Casla, I. M., Khodadadi, A. & Söder, L. Optimal day ahead planning and bidding strategy of battery storage unit participating in nordic frequency markets. *IEEE Access.* **10**, 76870–76883 (2022).

[2] Jain, A. & Gupta, S. Evaluation of electrical load demand forecasting using various machine learning algorithms. *Front. Energy Res.* **12**, 1408119 (2024).

[3] Suliman, M. S. & Farzaneh, H. Synthesizing the market clearing mechanism based on the national power grid using hybrid of deep learning and econometric models: evidence from the Japan Electric Power Exchange (JEPX) market. *J. Clean. Prod.* **411**, 137353 (2023).

[4] Chen, Y., Lin, C., Zhang, Y., Liu, J. & Yu, D. Day-ahead load forecast based on Conv2D-GRU_SC aimed to adapt to steep changes in load. *Energy* **302**, 131814 (2024).

[5] Debusschere, V. & Bacha, S. One week hourly electricity load forecasting using Neuro-Fuzzy and Seasonal ARIMA models. *IFAC Proc.* **45**, 97–102 (2012).

[6] Andersson, M. Modeling electricity load curves with hidden Markov models for demand-side management status estimation. *Int. Trans. Electr. Energy Syst.* **27** (3), e2265 (2017).

[7] MAa, T. Renewable energy generation effects on the electricity market: an empirical study on Japan’s Electricity Spot Market (Hunan Normal University, 2020).

[8] 柯宜均 & コイジュン Japan residential electricity consumption in response to climate change (2025).

[9] Misiurek, K., Olkuski, T. & Zyśk, J. Review of methods and models for forecasting electricity consumption. *Energies* **18**, 4032 (2025).

[10] Al-Shaikh, H., Rahman, M. A. & Zubair, A. Short-term electric demand forecasting for power systems using similar months approach based SARIMA. In *IEEE International Conference on Power, Electrical, and Electronics and Industrial Applications (PEEIACON)* 122–126 (IEEE, 2019).

[11] Andoh, P. Y. A., Sekyere, C. K. K., Mensah, L. D. & Dzebre, D. E. K. Forecasting electricity demand In Ghana with the Sarima model. *J. Appl. Eng. Technol. Sci. (JAETS)* **3**, 1–9 (2021).

[12] Kien, D. T., Huong, P. D. & Minh, N. D. Application of sarima model in load forecasting in Hanoi city. *Int. J. Energy Econ. Policy*. **13** (3), 164–170 (2023).

[13] Aksöz, A., Oyucu, S., Biçer, E. & Bayındır, R. Analysis of SARIMA models for forecasting electricity demand. In *12th International Conference on Smart Grid (icSmartGrid)* 767–771 (IEEE, 2024).

[14] Hernández, J. J., Zapirain, I., Camblong, H., Barroso, N. & Curea, O. Real implementation and testing of short-term building load forecasting: a comparison of SVR and NARX. *Energies* **18**, 1775 (2025).

[15] Anh, N. T. N. et al. Online SARIMA applied for short-term electricity load forecasting. *Appl. Intell.* **54** (1), 1003–1019 (2024).

[16] Mahajan, A., Das, S., Su, W. & Bui, V. H. Bayesian neural network-based approach for probabilistic prediction of building energy demands (2024).

[17] Zhang, J., Wang, J., Wang, R. & Hou, G. Forecasting next-day electricity prices with Hidden Markov Models. In *5th IEEE Conference on Industrial Electronics and Applications* 1736–1740 (IEEE, 2010).

[18] Erlwein, C., Benth, F. E. & Mamon, R. HMM filtering and parameter estimation of an electricity spot price model. *Energy Econ.* **32** (5), 1034–1043 (2010).

[19] Ullah, I., Ahmad, R. & Kim, D. A prediction mechanism of energy consumption in residential buildings using hidden markov model. *Energies* **11**, 358 (2018).

[20] Khetarpal, P., Nagpal, N., Kumar, M., Lakshmi, D. & Kassarwani, N. Short-term electricity load forecasting using modified hidden Markov model. In *International Conference on Renewable Power* 61–74 (Springer, 2023).

[21] Lu, S. & Bao, T. Short-term electricity load forecasting based on NeuralProphet and CNN-LSTM. *IEEE Access.* **12**, 76870–76879 (2024).

[22] Kong, W. et al. Short-term residential load forecasting based on LSTM recurrent neural network. *IEEE Trans. Smart Grid*. **10** (1), 841–851 (2017).

[23] Miah, M. S. U. et al. REDf: a deep learning model for short-term load forecasting to facilitate renewable integration and attaining the SDGs 7, 9, and 13. *PeerJ Comput. Sci.* **11**, e2819 (2025). 40567641 10.7717/peerj-cs.2819 PMC12190472

[24] Ahranjani, Y. K., Beiraghi, M. & Ghanizadeh, R. Short time load forecasting for Urmia city using the novel CNN-LTSM deep learning structure. *Electr. Eng.* **107** (1), 1253–1264 (2025).

[25] Kim, B. J. & Nam, I. W. A review of hybrid LSTM models in smart cities. *Processes* **13**, 2298 (2025).

[26] Hochreiter, S. The vanishing gradient problem during learning recurrent neural nets and problem solutions. *Int. J. Uncertain. Fuzziness Knowl.-Based Syst.* **6** (02), 107–116 (1998).

[27] Ye, H. et al. Multi-source data fusion-based grid-level load forecasting. *Applied Sciences* **15**, 4820 (2025).

[28] Kırat, O., Cicek, A. & Yerlikaya, T. A new artificial intelligence-based system for optimal electricity arbitrage of a second-life battery station in day-ahead markets. *Appl. Sci.* **14** (21), 10032 (2024).

[29] Kırat, O. & Çiçek, A. Artificial intelligence based optimal operation of green hydrogen refueling. In *2024 32nd Telecommunications Forum (TELFOR)* 1–4 (IEEE, 2024).

[30] Hong, N. N. & Duc, H. N. Virtual power plant’s optimal scheduling strategy in day-ahead and balancing markets considering reserve provision model of energy storage system. *Appl. Sci.* **14**, 2175 (2024).

[31] El-Azab, H. A. I., Swief, R., El-Amary, N. H. & Temraz, H. Seasonal forecasting of the hourly electricity demand applying machine and deep learning algorithms impact analysis of different factors. *Sci. Rep.* **15** (1), 9252 (2025). 40102500 10.1038/s41598-025-91878-0 PMC11920270

[32] Jiang, K. & Yamada, Y. A comprehensive analysis of imbalance signal prediction in the Japanese electricity market using machine learning techniques. *Energies* **18**, 2680 (2025).

[33] Muzaffar, S. & Afshari, A. Short-term load forecasts using LSTM networks. *Energy Procedia*. **158**, 2922–2927 (2019).

[34] IEA. Japan Electricity Security Policy [Online]. Available: https://www.iea.org/articles/japan-electricity-security-policy

[35] Hiruta, Y., Ishizaki, N. N., Ashina, S. & Takahashi, K. Regional and temporal variations in the impacts of future climate change on Japanese electricity demand: simultaneous interactions among multiple factors considered. *Energy Convers. Manage. X*. **14**, 100172 (2022).

[36] Otsuka, A. Regional energy demand in Japan: dynamic shift-share analysis. *Energy Sustain. Soc.* **6** (1), 10 (2016).

[37] Advisory, S. Everything you need to know about the Japanese Electricity Grid (Shulman, 2025). https://shulman-advisory.com/everything-you-need-to-know-about-the-japanese-electricity-grid/.

[38] Kyuhoshi Japan Travel & Culture Guide. https://www.kyuhoshi.com/map-of-japan/ (2025).

[39] Distribution, K. E. P. T. Kyushu Electric Power Transmission and Distribution ed (Ministry of Economy, Trade and Industry’s Agency for Natural Resources and Energy’s, 2025). https://www.kyuden.co.jp/td_area_jukyu/jukyu.html (2025).

[40] Distribution, K. E. P. T. Kansai electric power transmission and distribution. https://www.kansai-td.co.jp/denkiyoho/area-performance/past.html (2025).

[41] Distribution, H. E. P. T. Hokuriku electric power transmission and distribution. https://www.rikuden.co.jp/nw_jyukyudata/area_jisseki.html (2025).

[42] Distribution, H. E. P. T. Hokkaido electric power transmission and distribution. https://www.hepco.co.jp/network/con_service/public_document/supply_demand_results/index.html (2025).

[43] Network, C. E. P. Chugoku electric power network. https://www.energia.co.jp/nw/service/retailer/data/area/index.html (2025).

[44] Distribution, S. E. P. T. Shikoku electric power transmission and distribution. https://www.yonden.co.jp/nw/denkiyoho/download.html (2025).

[45] Grid, T. P. Tokyo electric power company Power Grid. https://www.tepco.co.jp/forecast/html/area_data-j.html (2025).

[46] Grid, C. E. P. Chubu electric power grid. https://powergrid.chuden.co.jp/denkiyoho/ (2025).

[47] Network, T. E. P. Tohoku electric power network. https://setsuden.nw.tohoku-epco.co.jp/download.html (2025).

[48] Ghasvarian Jahromi, K., Gharavian, D. & Mahdiani, H. R. Wind power prediction based on wind speed forecast using hidden Markov model. *J. Forecast.* **42** (1), 101–123 (2023).

[49] Kingma, D. P. & Ba, J. Adam: a method for stochastic optimization. *arXiv preprint arXiv:1412.6980* (2014).

[50] Goodfellow, I., Bengio, Y., Courville, A. & Bengio, Y. *Deep learning* (MIT press, 2016).

[51] Hyndman, R. J. & Athanasopoulos, G. *Forecasting: Principles and Practice* (OTexts, 2018).

[52] Mariko, P. C. & O’Neil, B. N. E. F. Power market outlook: East Meets West as Japan Prices Cool. https://about.bnef.com/insights/commodities/power-market-outlook-east-meets-west-as-japan-prices-cool/ (2025).

