# Supplementary material for: Impact of accurate load forecasting on electricity market stability in Japan using classical time-series and deep-learning methods
Source: Sci Rep. 2026 Apr 2;16:11781. doi: 10.1038/s41598-026-46859-2 (PMC13066410; doi:10.1038/s41598-026-46859-2)
Supplement: Supplementary file 1 — Supplementary Material 1 [file 41598_2026_46859_MOESM1_ESM.docx]

**Supplementary material for:**

**Impact of Accurate Load Forecasting on Electricity Market Stability in Japan Using Classical Time-Series and Deep-Learning Methods**

Dalia Rabie^1^, Mehran Moradi^1,4^, Wang Xuan^1^ and Hooman Farzaneh^1,2,3^*

*^1^Interdisciplinary Graduate School of Engineering Sciences, Kyushu University, Fukuoka 816-8580, Japan*

*^2^ Transdisciplinary Research and Education Center for Green Technologies, Kyushu University, Fukuoka, Japan*

*^3^Faculty of Engineering Sciences, Kyushu University, Fukuoka, Japan*

*^4^Department of Engineering and Physics, Karlstad University, 651 88 Karlstad, Sweden*

*Correspondence: [farzaneh.hooman.961@m.kyushu-u.ac.jp](mailto:farzaneh.hooman.961@m.kyushu-u.ac.jp)

**Section I: Theoretical Methodologies and Mathematical Frameworks**

This section dedicated exclusively to the theoretical underpinnings of the models employed. We examine the mathematical architectures, statistical assumptions, and algorithmic logic of three distinct forecasting paradigms

**1. Seasonal Autoregressive Integrated Moving Average (SARIMA)**

The SARIMA model is a powerful statistical method adept at analyzing and forecasting time-series data that exhibits clear seasonal patterns. Given that electricity demand follows strong daily and weekly cycles, SARIMA serves as a robust and interpretable baseline model.

- 1. **Formal Theoretical Definition**

The SARIMA model extends the conventional *ARIMA*$\left( p,d,q \right)$ Framework by explicitly incorporating seasonal components, resulting in $SARIMA\left( p,d,q \right)*\left( P,D,Q \right)s$ . The structure of the model is characterized by the following parameters:

- $\left( p,d,q \right)$: The non-seasonal orders for the Autoregressive (AR), Integrated (differencing), and Moving Average (MA) components.
- $\left( P,D,Q \right)$: The corresponding seasonal orders
- $s$: The seasonal period length. For this study, which uses hourly data with a distinct daily pattern, the seasonal period is set to s = 24.
  1. **Mathematical Formulation and Mechanism**

The complete SARIMA model using the backshift operator $\boldsymbol{B}$, where $\boldsymbol{B}^{\boldsymbol{k}}$*****$\boldsymbol{D}_{\boldsymbol{t}}\boldsymbol{=}\boldsymbol{D}_{\boldsymbol{t-k}}$**.** is mathematically expressed as [1]:

| $\boldsymbol{\phi}_{\boldsymbol{p}}\left( \boldsymbol{B} \right){\boldsymbol{\Phi}_{\boldsymbol{P}} \left( \boldsymbol{B} \right)}^{\boldsymbol{S}}\left( \boldsymbol{1-B} \right)^{\boldsymbol{d}}\left( \boldsymbol{1-}\boldsymbol{B}^{\boldsymbol{s}} \right)^{\boldsymbol{D}}\boldsymbol{*}\boldsymbol{D}_{\boldsymbol{t}}\boldsymbol{=}\boldsymbol{\theta}_{\boldsymbol{q}}\left( \boldsymbol{B} \right){\boldsymbol{\Theta}_{\boldsymbol{Q}}\left( \boldsymbol{B} \right)}^{\boldsymbol{S}}$) $\boldsymbol{\varepsilon}_{\boldsymbol{t}}$ | **(S1)** |
| --- | --- |

where the components are defined as:

1. Autoregressive Component $\boldsymbol{\phi}_{\boldsymbol{p}}\left( \boldsymbol{B} \right){\boldsymbol{\Phi}_{\boldsymbol{P}} \left( \boldsymbol{B} \right)}^{\boldsymbol{S}}$: This term models the dependency of the current observation $\boldsymbol{D}_{\boldsymbol{t}}$ on past values. It combines the non-seasonal $\boldsymbol{\phi}_{\boldsymbol{p}}\left( \boldsymbol{B} \right)$ and seasonal ${\boldsymbol{\Phi}_{\boldsymbol{P}} \left( \boldsymbol{B} \right)}^{\boldsymbol{S}}$ AR operators:

| $\boldsymbol{\phi}_{\boldsymbol{p}}\left( \boldsymbol{B} \right){\boldsymbol{\Phi}_{\boldsymbol{P}} \left( \boldsymbol{B} \right)}^{\boldsymbol{S}}\boldsymbol{=(1-}\boldsymbol{\phi}_{\boldsymbol{1}}\boldsymbol{B}^{\boldsymbol{1}}\boldsymbol{-}\boldsymbol{\phi}_{\boldsymbol{2}}\boldsymbol{B}^{\boldsymbol{2}}\boldsymbol{-\cdot\cdot\cdot\cdot\cdot\cdot\cdot\cdot\cdot}\boldsymbol{-\phi}_{\boldsymbol{p}}\boldsymbol{B}^{\boldsymbol{p}}\mathbf{)}\boldsymbol{(1-}\boldsymbol{\Phi}_{\boldsymbol{1}}\boldsymbol{B}^{\boldsymbol{S}}\boldsymbol{-}\boldsymbol{\Phi}_{\boldsymbol{2}}\boldsymbol{B}^{\boldsymbol{2}\boldsymbol{S}}\boldsymbol{-\cdot\cdot\cdot\cdot\cdot\cdot}{\boldsymbol{-}\boldsymbol{\Phi}}_{\boldsymbol{P}}\boldsymbol{B}^{\boldsymbol{PS}}\mathbf{)}$ | **(S2)** |
| --- | --- |

2. Integrated (I) Component $\left( \boldsymbol{1-B} \right)^{\boldsymbol{d}}\left( \boldsymbol{1-}\boldsymbol{B}^{\boldsymbol{s}} \right)^{\boldsymbol{D}}$: This component is applied to render the time series stationary by removing trends and seasonality. The terms d and D represent the number of non-seasonal and seasonal differences applied, respectively.

3. Moving Average Component $\boldsymbol{\theta}_{\boldsymbol{q}}\left( \boldsymbol{B} \right) {\boldsymbol{\Theta}_{\boldsymbol{Q}}\left( \boldsymbol{B} \right)}^{\boldsymbol{S}}$: This term models the dependency of the current observation on past forecast errors $\boldsymbol{\varepsilon}_{\boldsymbol{t}}$. It combines the non-seasonal $\boldsymbol{\theta}_{\boldsymbol{q}}\left( \boldsymbol{B} \right)$ and seasonal ${\boldsymbol{\Theta}_{\boldsymbol{Q}}\left( \boldsymbol{B} \right)}^{\boldsymbol{S}}$ MA operators[1]:

| $\boldsymbol{\theta}\left( \boldsymbol{B} \right){\boldsymbol{\Theta}\left( \boldsymbol{B} \right)}^{\boldsymbol{S}}\boldsymbol{\varepsilon}_{\boldsymbol{t}}\boldsymbol{=(1-}\sum_{\boldsymbol{i=1}}^{\boldsymbol{q}} \boldsymbol{\theta}_{\boldsymbol{i}}\boldsymbol{B}^{\boldsymbol{i}}$**)** $\boldsymbol{(1-}\sum_{\boldsymbol{i=1}}^{\boldsymbol{Q}} \boldsymbol{\Theta}_{\boldsymbol{i}}\boldsymbol{B}^{\boldsymbol{iS}}$**)*** $\boldsymbol{\varepsilon}_{\boldsymbol{t}}$ | **(S3)** |
| --- | --- |

Error Term ($\boldsymbol{\varepsilon}_{\boldsymbol{t}}$): This term represents the residual error at time t. A core assumption of the model is that $\boldsymbol{\varepsilon}_{\boldsymbol{t}}$ is a white noise process, characterized by a zero mean, constant variance ($\boldsymbol{\sigma}^{\boldsymbol{2}}$), and no serial autocorrelation.

As shown in Figure 1, the SARIMA model implementation follows the Box–Jenkins methodology, which involves five key steps. First, the stationarity of the time series is verified using the Augmented Dickey–Fuller (ADF) test, and differencing is applied until stationarity is achieved, determining parameters d and D. Next, parameter identification is conducted using the autocorrelation (ACF) and partial autocorrelation (PACF) plots to identify possible AR and MA orders (p, q, P, Q). In the model estimation and selection stage, multiple candidate models are fitted, and the optimal one is chosen based on the Akaike Information Criterion (AIC), balancing model fit and complexity to avoid overfitting. Diagnostic checking then ensures that residuals resemble white noise through the Ljung–Box test and normality assessments such as Q–Q plots. Finally, once validated, the model is applied to out-of-sample forecasting to generate reliable short-term demand predictions.


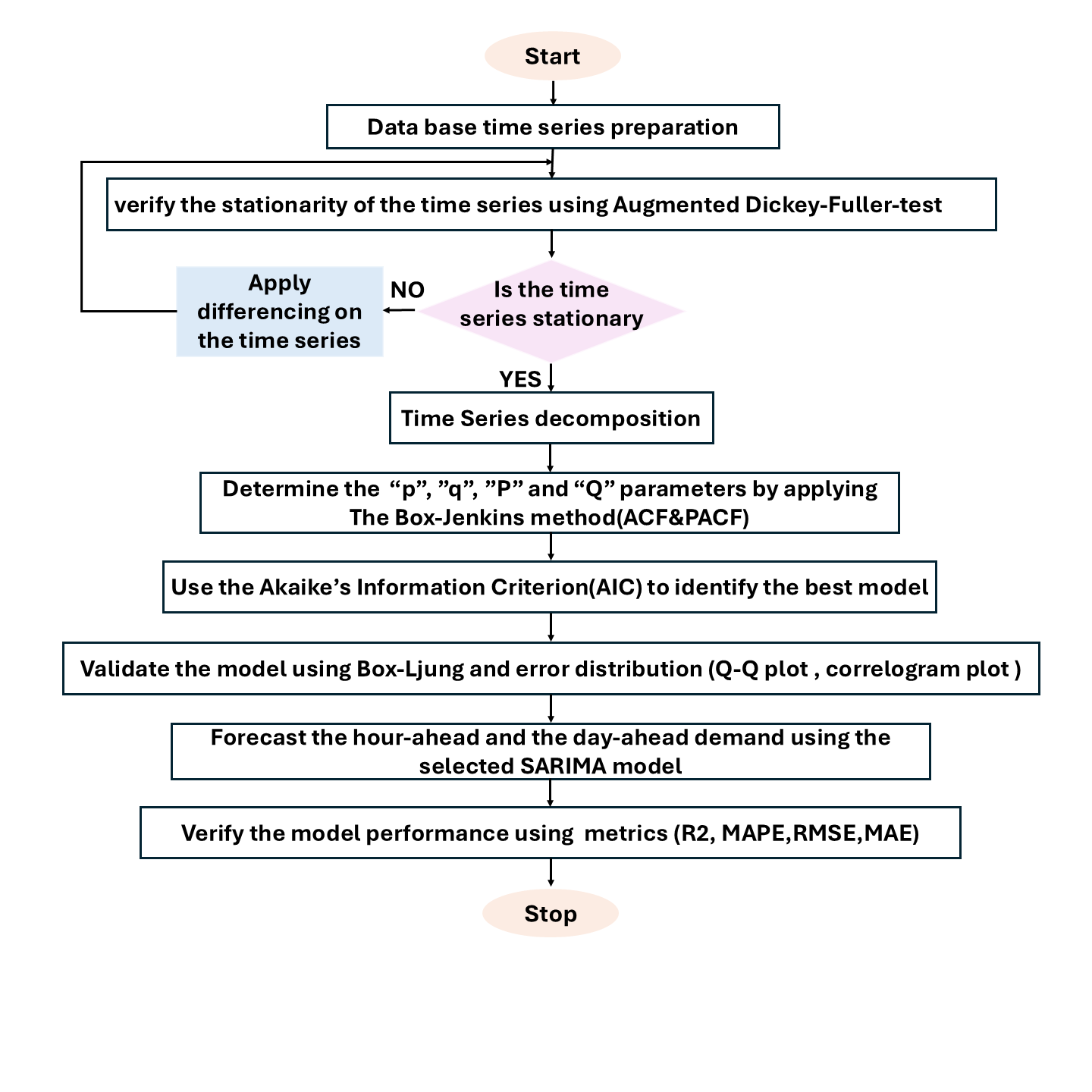


**Fig. 1 SARIMA Forecasting Flowchart**

**2. Hidden Markov Model (HMM)**

The HMM is a probabilistic model that describes a system as a Markov process with unobserved (hidden) states. For electricity demand, these hidden states can be interpreted as distinct consumption regimes (e.g., low, medium, high load). The model assumes that the observation at any given time is generated by a probability distribution corresponding to the current hidden state [2].

**2.1 Theoretical Architecture**

A continuous observation HMM is formally defined by the parameter set $\lambda=(A,B,\pi)$, where:

1. **Initial State Distribution** $\boldsymbol{(\pi)}$: A vector representing the probability of the system starting in each hidden state $\theta i$.

| $\pi_{i}=P\left( q_{t}=\theta_{i} \right), 1\leq i\leq N$ | **(S4)** |
| --- | --- |

Where

| $\sum_{i=1}^{N} \pi_{i}=1$ | **(S5)** |
| --- | --- |

1. **State Transition Probability Matrix** $\boldsymbol{(A)}$**:** An $N\times N$ Matrix where each element $aij$ represents the probability of transitioning from state $\theta i$ to state $\theta j$.

| $a_{ij}=P\left( q_{t+1}=\frac{\theta_{j}}{q_{t}}=\theta_{i} \right), 1\leq i, j\leq N$ | **(S6)** |
| --- | --- |

where each row must sum to one:

| $a_{ij}>0, \sum_{i=1}^{N} a_{ij}=1$ | **(S7)** |
| --- | --- |

1. **Observation Probability Distribution (B):** A set of probability distributions, one for each hidden state. For continuous observations like electricity load, each state's emission probability is modelled by a Gaussian distribution with mean $\mu_{i}$ and variance $\sigma_{i}^{2}$:

| $b_{i}\left( k \right)=P\left( o_{t}=\frac{d_{k}}{q_{t}}=\theta_{i} \right), 1\leq i\leq N, 1\leq k\leq M$ | **(S8)** |
| --- | --- |
| $b_{i}\left( k \right)=\frac{1}{\sqrt{2\pi\sigma_{i}^{2}}}\exp\left( -\frac{\left( d_{k}-\mu_{i} \right)^{2}}{2\sigma_{i}^{2}} \right)$ | **(S9)** |

Each observation distribution must also satisfy:

| $b_{i}\left( k \right)\geq0, \sum_{k=1}^{M} b_{i}\left( k \right)=1$ | **(S10)** |
| --- | --- |

**2.2 Algorithmic Implementation**

Figure 2 shows the implementation framework of the HMM for electricity demand forecasting. The process begins with historical electricity demand data, which is used for model training via the Baum–Welch algorithm. At this stage, the optimal number of hidden states is identified, and the model estimates the state transition probabilities and observation probabilities accordingly. The trained model then undergoes evaluation and diagnostic checking to ensure statistical adequacy. If the model fails to meet performance criteria, it is retrained with adjusted parameters; otherwise, it proceeds to the forecasting phase. In this phase, the Forward algorithm is used to calculate likelihoods, while the Viterbi algorithm estimates the most probable hidden state sequence. The resulting outputs are then used to generate accurate electricity demand forecasts, completing the iterative modelling process.

. To balance model accuracy and parsimony, information criteria are applied that penalize excessive parameters (p). The two commonly used measures are the Akaike Information Criterion (AIC) and the Bayesian Information Criterion (BIC), defined respectively as [2]:

| $\mathrm{AIC}=-2\log L+2p$ | **(S11)** |
| --- | --- |

| $\mathrm{BIC}=-2\log L+p\log T$ | **(S12)** |
| --- | --- |

where $L$ is the maximum likelihood of the model and $T$ is the number of observations. The model with the lowest AIC or BIC value is selected as the optimal representation of the underlying system dynamics.


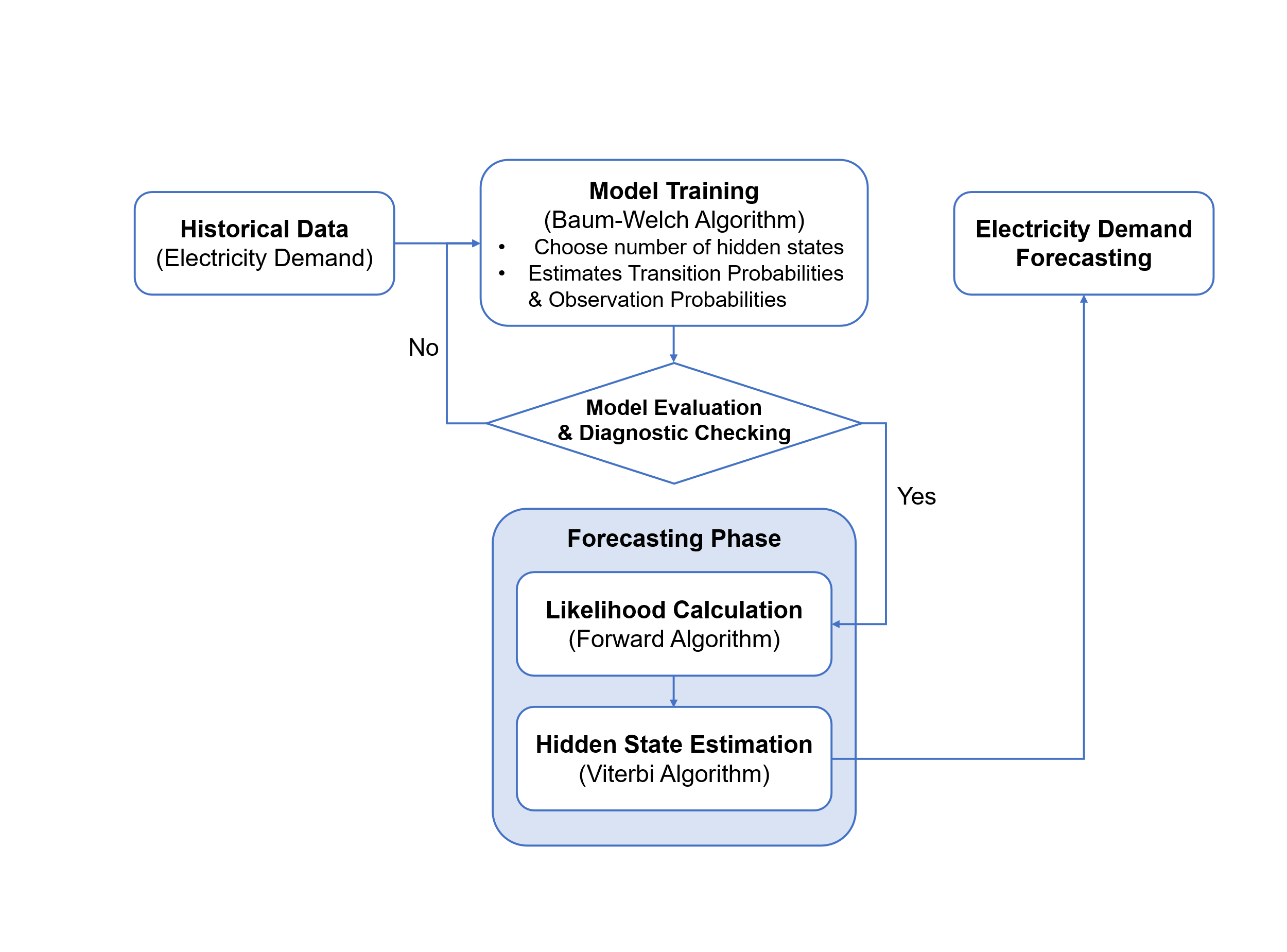


**Fig.2 HMM Forecasting flowchart**

**3. Long Short-Term Memory (LSTM) Network**

The LSTM network is composed of a cell state and three main gate units, as shown in Figure 4. The cell state acts as the memory of the network and allows it to store and transfer useful information across long periods within a sequence. The three gates, known as forget, input, and output gates, control how information flows through the network by using sigmoid activation functions and multiplication operations. These gating mechanisms allow the model to selectively retain or discard information over time, thereby mitigating the vanishing and exploding gradient problems commonly encountered in conventional recurrent neural networks. As a result, the LSTM network can better capture and represent long term dependencies in time series data[3].

**
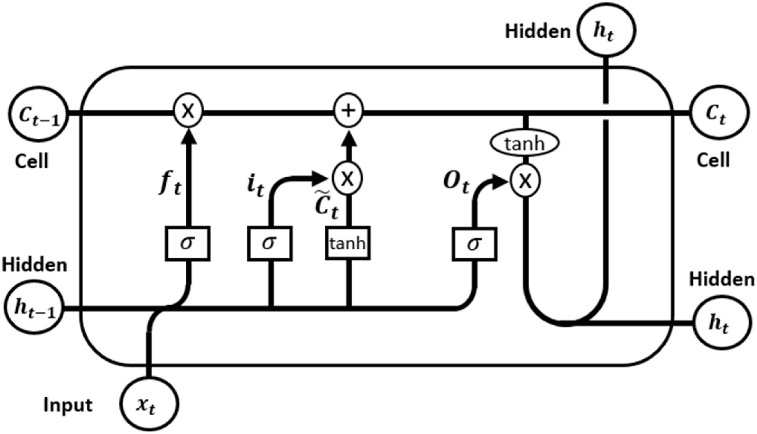
**

**Fig 4: LSTM Flowchart [4]**

**3.1 The Gating and Optimization Theory**

Based on the LSTM network architecture illustrated in Figure 4, the forget gate is responsible for removing unnecessary information from the cell state. It takes into account the current input $x_{t}$, and the previous hidden state $h_{t-1}$ and produces a forget ratio $f_{t}$ within the range [0,1] using a sigmoid activation function. This ratio determines how much of the past information should be retained or discarded at each time step. The input gate plays an essential role in deciding what new information should enter the cell state. It operates through two coordinated parts. The first part applies a sigmoid activation function to produce a selection ratio $i_{t}$, which determines the extent to which the current input contributes to updating the memory. The second part generates a set of candidate values $C_{t}$ using a hyperbolic tangent activation function, representing potential new information that could be added to the cell state. The outputs of these two processes are then merged with the previous cell state$C_{t-1}$ and the effect of the forget gate to form the updated cell state $C_{t}$. Through this process, the LSTM effectively balances newly received data with retained past information, allowing it to learn long-term dependencies more efficiently. The final component, known as the output gate, determines how much information from the cell state should be passed on as output. It first calculates a selection ratio $O_{t}$ through a sigmoid activation function, which controls the extent of information to be revealed. The cell state is then transformed using a hyperbolic tangent activation function, and the resulting values are multiplied by $O_{t}$ to generate the current hidden state $h_{t}$. This mechanism enables the LSTM network to selectively expose useful information from its internal memory at each time step while preserving the rest for future use. The detailed process is elaborated as follows [4]:

| $i_{t}=\sigma(W_{i}.\left[ h_{t-1},x_{t} \right]+b_{i})$ | **(S13)** |
| --- | --- |
| $f_{t}=\sigma(W_{f}.\left[ h_{t-1},x_{t} \right]+b_{f}))$ | **(S14)** |
| $\tilde{C}_{t}=tanh(W_{c}.\left[ h_{t-1},x_{t} \right])$ | **(S15)** |
| $C_{t}=f_{t}.C_{t-1}+i_{t}.\tilde{C}_{t}$ | **(S16)** |
| $O_{t}=\sigma(W_{O}.\left[ C_{t},h_{t-1},x_{t} \right]+b_{O})$ | **(S17)** |
| $h_{t}=O_{t}.tanh(C_{t})$ | **(S18)** |

Where $x_{t}$ represents the input vector at time step $t$, while $h_{t-1}$ and $C_{t-1}$ correspond to the hidden and cell states carried over from the previous time step. The three gating components, the input gate ($i_{t}$), forget gate ($f_{t}$) and output gate ($O_{t}$), regulate the flow of information within the network. The sigmoid activation function $\sigma(.)$ determines how much information passes through each gate, and the hyperbolic tangent function $tanh(.)$ introduces nonlinearity that enables the network to represent complex temporal patterns. Each gate contains its own trainable parameters, the weight matrices ($W_{*}$) and bias terms ($b_{*}$), which are optimized during the training process to reduce forecasting error. Through these mechanisms, the LSTM can decide what information to retain or discard from past sequences, making it suitable for learning long-term dependencies in time series such as electricity demand.

**Section 2: Technical Details, Parameters, and Tuned Values**

Having established the theoretical frameworks, this section details the specific technical implementation of these models within the study. It covers the parameter tuning, layer configurations, and specific coefficient values derived for the nine regional power grids of Japan

**1.** **SARIMA Technical Configuration and Tuning Results**

Following the rigorous application of the Box–Jenkins methodology outlined in Figure 1, specifically the iterative process of .differencing and order identification based on AIC minimization, the optimal model architectures were established for each of the nine regions. The resulting model specifications, including the identified orders $\left( p,d,q \right) and \left( P,D,Q \right)_{S}$, along with their performance metrics, are detailed in Table.1. Furthermore, the specific coefficient values for the autoregressive and moving average terms, derived during the estimation phase to minimize the error term $\boldsymbol{\varepsilon}_{\boldsymbol{t}}$, are presented in Table 2.

Table 1: The SARIMA Model for each region, along with the metrics

| **Region** | Model | MAE | RMSE | MAPE | R2 | Ljung-Box Q (Lag 1) | Ljung-Box P-value (Lag 1) |
| --- | --- | --- | --- | --- | --- | --- | --- |
| Hokkaido | (0,1,5)(0,0,2)24 | 55 | 70.3 | 1.7 | 0.98 | 0.21 | 0.64 |
| Tohoku | (5,1,2)(0,0,2)24 | 123.93 | 172.7 | 1.37 | 0.98 | 0.78 | 0.38 |
| Tokyo | (2,1,2)(0,0,2)24 | 408.2 | 575.1 | 1.28 | 0.99 | 0.04 | 0.85 |
| Chubu | (1,1,4)(1,0,2)24 | 165.8 | 233.7 | 1.16 | 0.99 | 0.03 | 0.86 |
| Hokuriku | (0,1,5)(0,0,2)24 | 48.2 | 65.7 | 1.5 | 0.98 | 0.01 | 0.92 |
| Kansai | (0,1,5)(1,0,2)24 | 173.2 | 211.9 | 1.33 | 0.93 | 0.69 | 0.41 |
| Chugoku | (1,1,0)(0,0,2)24 | 119.7 | 160.6 | 1.8 | 0.97 | 0.16 | 0.69 |
| Shikoku | (5,1,1)(0,0,2)24 | 58.9 | 79.44 | 1.9 | 0.98 | 0.23 | 0.63 |
| Kyushu | (1,1,2)(1,0,2)24 | 103.3 | 144.8 | 1.09 | 0.99 | 0.79 | 0.37 |

Table 2: The tuning parameter for each region's model.

| Hokkaido | | | |  | **Tohoku** | | | |
| --- | --- | --- | --- | --- | --- | --- | --- | --- |
| Parameter | Coefficient | Std. Error | P Value |  | **Parameter** | **Coefficient** | **Std. Error** | **P Value** |
| MA.L1 | 0.351861 | 0.004428 | 0 |  | AR.L1 | 0.606695 | 0.019453 | 1.5E-213 |
| MA.L2 | 0.161922 | 0.005177 | 1E-214 |  | AR.L2 | 0.725276 | 0.025149 | 7E-183 |
| MA.L3 | 0.002738 | 0.005482 | 0.617485 |  | AR.L3 | -0.2618 | 0.008567 | 4.2E-205 |
| MA.L4 | -0.16856 | 0.005224 | 1.9E-228 |  | AR.L4 | -0.25132 | 0.005945 | 0 |
| MA.L5 | -0.02967 | 0.004877 | 1.17E-09 |  | AR.L5 | 0.073597 | 0.006067 | 7.25E-34 |
| MA.S.L24 | 0.698471 | 0.004401 | 0 |  | MA.L1 | -0.27233 | 0.019099 | 3.94E-46 |
| MA.S.L48 | 0.395331 | 0.004355 | 0 |  | MA.L2 | -0.73619 | 0.019263 | 0 |
|  | | | |  | MA.S.L24 | 0.848133 | 0.004009 | 0 |
|  |  |  |  |  | MA.S.L48 | 0.453785 | 0.003944 | 0 |

| **Tokyo** | | | |  | **Chubu** | | | |
| --- | --- | --- | --- | --- | --- | --- | --- | --- |
| **Parameter** | **Coefficient** | **Std. Error** | **P Value** |  | **Parameter** | **Coefficient** | **Std. Error** | **P Value** |
| AR.L1 | 1.683359 | 0.004129 | 0 |  | AR.L1 | -0.57753 | 0.019137 | 4.5E-200 |
| AR.L2 | -0.74342 | 0.004025 | 0 |  | MA.L1 | -33.032 | 19.77905 | 0.09491 |
| MA.L1 | -0.95392 | 0.005966 | 0 |  | MA.L2 | -37.6393 | 22.24047 | 0.090574 |
| MA.L2 | -0.03976 | 0.005971 | 2.75E-11 |  | MA.L3 | -25.6191 | 14.8067 | 0.083588 |
| MA.S.L24 | 0.822378 | 0.003891 | 0 |  | MA.L4 | -15.9101 | 9.115187 | 0.080906 |
| MA.S.L48 | 0.424577 | 0.003843 | 0 |  | AR.S.L24 | 0.998329 | 0.000217 | 0 |
|  | | | |  | MA.S.L24 | -0.50278 | 0.003539 | 0 |
|  |  |  |  |  | MA.S.L48 | -0.38285 | 0.003709 | 0 |

| **Hokuriku** | | | |  | **Kansai** | | | |
| --- | --- | --- | --- | --- | --- | --- | --- | --- |
| Parameter | Coefficient | Std. Error | P Value |  | **Parameter** | **Coefficient** | **Std. Error** | **P Value** |
| MA.L1 | 0.548907 | 0.003883 | 0 |  | MA.L1 | 0.540252 | 0.003659 | 0 |
| MA.L2 | 0.3422 | 0.005043 | 0 |  | MA.L2 | 0.404951 | 0.004762 | 0 |
| MA.L3 | 0.098387 | 0.005775 | 4.47E-65 |  | MA.L3 | 0.235645 | 0.005193 | 0 |
| MA.L4 | -0.16577 | 0.005492 | 3.8E-200 |  | MA.L4 | -0.07201 | 0.005244 | 6.62E-43 |
| MA.L5 | 0.073677 | 0.004615 | 2.32E-57 |  | MA.L5 | 0.073506 | 0.00512 | 9.63E-47 |
| MA.S.L24 | 0.832025 | 0.004232 | 0 |  | AR.S.L24 | 0.997896 | 0.000271 | 0 |
| MA.S.L48 | 0.426199 | 0.004147 | 0 |  | MA.S.L24 | -0.54552 | 0.00336 | 0 |
|  | | | |  | MA.S.L48 | -0.31299 | 0.00355 | 0 |

| **Chugoku** | | | |  | **Shikoku** | | | |
| --- | --- | --- | --- | --- | --- | --- | --- | --- |
| Parameter | Coefficient | Std. Error | P Value |  | **Parameter** | **Coefficient** | **Std. Error** | **P Value** |
| AR.L1 | 0.443531 | 0.003663 | 0 |  | AR.L1 | 0.03243 | 0.01983 | 0.101968 |
| MA.S.L24 | 0.772597 | 0.004395 | 0 |  | AR.L2 | 0.221062 | 0.010166 | 7.5E-105 |
| MA.S.L48 | 0.412677 | 0.004213 | 0 |  | AR.L3 | -0.03451 | 0.00517 | 2.47E-11 |
|  | | | |  | AR.L4 | -0.17562 | 0.004494 | 0 |
|  |  |  |  |  | AR.L5 | 0.154189 | 0.0051 | 8.6E-201 |
|  |  |  |  |  | MA.L1 | 0.474883 | 0.020015 | 1.9E-124 |
|  |  |  |  |  | MA.S.L24 | 0.756043 | 0.003953 | 0 |
|  |  |  |  |  | MA.S.L48 | 0.420154 | 0.003842 | 0 |

| **Kyushu** | | | |
| --- | --- | --- | --- |
| **Parameter** | **Coefficient** | **Std. Error** | **P Value** |
| AR.L1 | 0.299751 | 0.014336 | 4.49E-97 |
| MA.L1 | 0.16553 | 0.014238 | 3.04E-31 |
| MA.L2 | 0.178905 | 0.007258 | 3.7E-134 |
| AR.S.L24 | 0.996891 | 0.000414 | 0 |
| MA.S.L24 | -0.62831 | 0.003618 | 0 |
| MA.S.L48 | -0.22198 | 0.003718 | 0 |

**2. Hidden Markov Model (HMM) Technical Details**

In HMM forecasting model, several model parameters and technical settings were adjusted to ensure stable convergence and reliable predictive performance. The key parameters include the number of hidden states, which represents distinct demand patterns observed in the data, the form of the emission distribution used to model the continuous load values, the state transition probabilities and the initial state distribution as shown in equation (S4) to (S10). These parameters were estimated from the training data using the Baum-Welch algorithm.

The model follows the standard two-layer structure of an HMM, consisting of a latent state layer and an emission layer. No additional hierarchical or stacked structures were introduced. Several technical settings were also tuned, such as the maximum number of iterations is set as 500, the type of covariance matrix used in the emission distribution is diagonal, the scaling method is “StandardScaler” which is a method that average is 0 and variance is 1.

This research tested the number of states m from 16 to 20. The results of AIC and BIC (equations S11 and S12 in the supplementary file) obtained by HMM with different numbers of states m are shown in Figure 3. HMM gave better results for m=18 in Kyushu, m=19 in Chubu, Hokuriku, Tokyo, and m=20 in Chugoku, Hokkaido, Kansai, Shikoku and Tohoku.


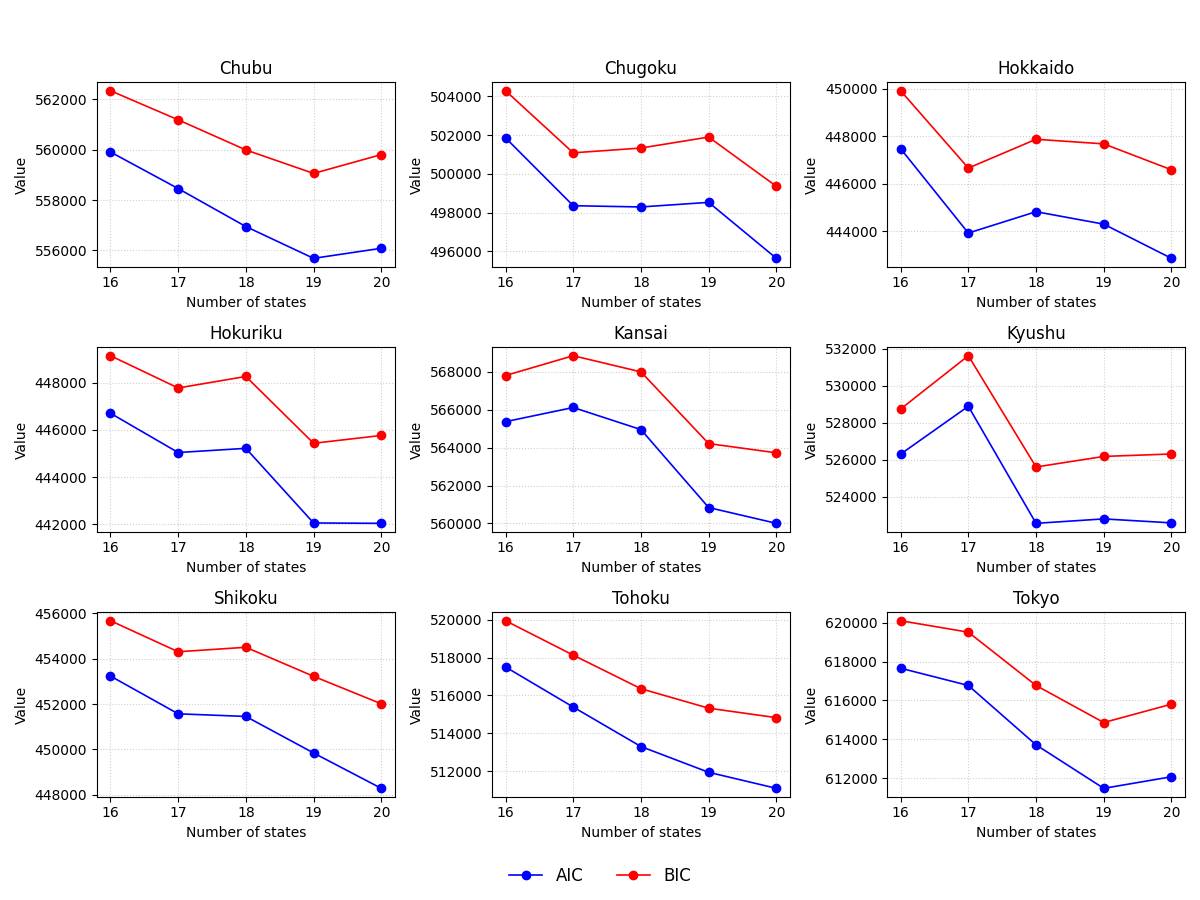


Fig. 3 AIC and BIC values for different numbers of states

**3.** **LSTM Technical Configuration and Hyperparameters**

The LSTM models were built using a stacked architecture. Two distinct models were trained: a Day-Ahead model and an Hour-Ahead model. The technical specifications reveal a preference for deep sequence learning.

The LSTM hyper-parameters used for the day-ahead and hour-ahead forecasting models are summarized in Table 3.

**Table *3*: The tuning parameter for each region's model.**

| **Parameter** | **Day-Ahead Model** | **Hour-Ahead Model** |
| --- | --- | --- |
| **Forecast horizon** | 24 Hours | 1 Hour |
| **Input sequence length** | 144 Hours | 24 Hours |
| **Train/Test split** | 80%/20% | 80%/20% |
| **Scaling method** | MinMaxScalar (0-1) | MinMaxScalar (0-1) |
| **Batch size** | 32 | 32 |
| **Epochs** | 500 | 500 |
| **Early stopping** | Patience = 100, restore best | Patience = 20, restore best |
| **Optimizer** | Adam | Adam |
| **Loss function** | MSE | MSE |
| **Model architecture depth** | 2 LSTM + 1 Dense + Output | 2 LSTM + 1 Dense + Output |
| **LSTM layer 1 units** | 128 | 128 |
| **LSTM layer 2 units** | 64 | 64 |
| **Dense hidden layer units** | 64 (ReLU) | 64 (ReLU) |
| Output layer | 24 (Linear) | 1 (Linear) |
| Input shape | (144, 1) | (24, 1) |
| Prediction output shape | (24,) | (1,) |

[1] Andoh PYA, Sekyere CKK, Mensah LD, Dzebre DEK. Forecasting Electricity Demand In Ghana With The Sarima Model. Journal of Applied Engineering and Technological Science (JAETS). 2021;3:1 - 9.

[2] Ghasvarian Jahromi K, Gharavian D, Mahdiani HR. Wind power prediction based on wind speed forecast using hidden Markov model. Journal of Forecasting. 2023;42:101-23.

[3] Goodfellow I, Bengio Y, Courville A, Bengio Y. Deep learning: MIT press Cambridge; 2016.

[4] Suliman MS, Farzaneh H. Synthesizing the market clearing mechanism based on the national power grid using hybrid of deep learning and econometric models: evidence from the Japan Electric Power Exchange (JEPX) market. Journal of Cleaner Production. 2023;411:137353.
